# Supplementary material for: Systematic review of the effectiveness of selected drugs for preventive chemotherapy for Taenia solium taeniasis
Source: PLoS Negl Trop Dis. 2020 Jan 16;14(1):e0007873. doi: 10.1371/journal.pntd.0007873 (PMC6964831; doi:10.1371/journal.pntd.0007873)
Supplement: S1 File — (DOCX) [file pntd.0007873.s002.docx]

# S1 File. Search strategies and results

## Search terms used when searching for studies

| Keyword area | Search terms | Search fields | Notes |
| --- | --- | --- | --- |
| 1. Condition | Taenia solium OR Taenia | [MESH] and ([Title/Abstract] or [Text word]) |  |
| 1. Intervention | albendazole OR niclosamide OR praziquantel OR anthelmintics OR chemoprevention OR mass drug administration OR deworm* | [MESH] ([Title/Abstract] or [Text word]) | deworm* is only searched in [Title/Abstract] or [Text word] as it is not a MESH term |
| 1. Publication type | Case reports OR Editorial | [publication type] | To exclude clearly non-relevant publication types |

Variations of the search terms for the condition and intervention keyword areas were also searched in Spanish, French and Portuguese – where the search engine supported foreign characters.

Keyword areas were combined using AND or NOT: (1 AND 2) NOT 3.

## Search results for each source

| Database | Keyword areas searched | Date searched | No. refs found | No. refs after duplicates removed | Potentially meets the inclusion criteria | Include |
| --- | --- | --- | --- | --- | --- | --- |
| Electronic Databases |  |  |  |  |  |  |
| PubMed (NLM) | (1 AND 2) NOT 3 | 26/9/18 | 609 | 608 | 70 | 22 |
| Embase (Ovid) | (1 AND 2) NOT 3 | 26/9/18 | 1580 | 1201 | 78 | 8 |
| LILACS (BVSalud) | 1 AND 2 | 26/9/18 | 111 | 103 | 6 | 0 |
| SciELO | 1 AND 2 | 26/9/18 | 32 | 14 | 1 | 0 |
| Cochrane CENTRAL | 1 |  | 69 | 46 | 6 | 0 |
| CAB Abstracts | 1 AND 2 | 26/9/18 | 2005 | 1495 | 71 | 3 |
| Specialized sources of systematic reviews |  |  |  |  |  |  |
| Cochrane Database of Systematic Reviews | 1 | 26/9/18 | 1 | 1 | 0 | 0 |
| Database of Abstracts of Reviews of Effects (DARE) | 1 | 26/9/18 | 0 | 0 | 0 | 0 |
| Health Technology Assessment (HTA) | 1 | 26/9/18 | 0 | 0 | 0 | 0 |
| NHS Economic Evaluation Database | 1 | 26/9/18 | 1 | 1 | 1 | 0 |
| Epistemonikos – SRs and primary studies | 1 | 26/9/18 | 94 | 63 | 0 | 0 |
| Total: |  |  | 4503 | 3532 | 233 | 33 |
| Extra searches  3ie - International Initiative for Impact Evaluation; Google and Google Scholar; System for Information on Grey Literature in Europe (Open grey – [www.opengrey.eu](http://www.opengrey.eu)); WHO ICTRP, reference list of included studies; reference list of systematic reviews; reference list of key WHO/PAHO documents, experts. | 1 (AND 2) |  |  | 23 | 23 | 2 |
| TOTAL: |  |  |  | 3555 | 256 | 35 |

## Search strategies

**PubMED (NLM)** – 30 July 2018

| Search | Query | Items found |
| --- | --- | --- |
| #11 | Search #9 NOT #10 | 605 |
| #10 | Search ("case reports"[Publication Type]) OR "editorial"[Publication Type] | 2350549 |
| #9 | Search #5 AND #8 | 790 |
| #8 | Search #6 or #7 | 56164 |
| #7 | Search ((((((((((((((albendazol*[Text Word]) OR niclosamid*[Text Word]) OR praziquantel[Text Word]) OR prazicuantel[Text Word]) OR anthelminti*[Text Word]) OR antihelminti*[Text Word]) OR chemoprevention[Text Word]) OR quimiopreven*[Text Word]) OR chimioprevention[Text Word]) OR mass drug administration[Text Word]) OR administración masiva de medicamentos[Text Word]) OR administration massive de médicaments[Text Word]) OR distribution massive de médicaments[Text Word]) OR administração massiva dos medicamentos[Text Word]) OR deworm*[Text Word] | 39794 |
| #6 | Search (((((albendazole[MeSH Terms]) OR niclosamide[MeSH Terms]) OR praziquantel[MeSH Terms]) OR anthelmintics[MeSH Terms]) OR chemoprevention[MeSH Terms]) OR "mass drug administration"[MeSH Terms] | 40910 |
| #5 | Search #3 OR #4 | 7374 |
| #4 | Search ((((taenia solium[Text Word]) OR taenia[Text Word]) OR teniasis[Text Word]) OR teniase[Text Word]) | 7001 |
| #3 | Search (taenia solium[MeSH Terms]) OR Taenia[MeSH Terms] | 3866 |

**EMBASE (Ovid)** – 30 July 2018

Database: Embase Classic+Embase <1947 to 2018 Week 31>

Search Strategy:

--------------------------------------------------------------------------------

1 exp Taenia solium/ (2494)

2 exp Taenia/ (6055)

3 taenia solium.mp. [mp=title, abstract, heading word, drug trade name, original title, device manufacturer, drug manufacturer, device trade name, keyword, floating subheading word, candidate term word] (3192)

4 taenia.mp. [mp=title, abstract, heading word, drug trade name, original title, device manufacturer, drug manufacturer, device trade name, keyword, floating subheading word, candidate term word] (9196)

5 teniasis.mp. [mp=title, abstract, heading word, drug trade name, original title, device manufacturer, drug manufacturer, device trade name, keyword, floating subheading word, candidate term word] (114)

6 teniase.mp. [mp=title, abstract, heading word, drug trade name, original title, device manufacturer, drug manufacturer, device trade name, keyword, floating subheading word, candidate term word] (16)

7 1 or 2 or 3 or 4 or 5 or 6 (9269)

8 exp albendazole/ (13114)

9 exp niclosamide/ (1802)

10 exp praziquantel/ (8897)

11 exp anthelmintic agent/ (125609)

12 exp chemoprophylaxis/ (23839)

13 albendazol*.mp. [mp=title, abstract, heading word, drug trade name, original title, device manufacturer, drug manufacturer, device trade name, keyword, floating subheading word, candidate term word] (13835)

14 niclosamid*.mp. [mp=title, abstract, heading word, drug trade name, original title, device manufacturer, drug manufacturer, device trade name, keyword, floating subheading word, candidate term word] (1936)

15 praziquantel.mp. [mp=title, abstract, heading word, drug trade name, original title, device manufacturer, drug manufacturer, device trade name, keyword, floating subheading word, candidate term word] (9530)

16 prazicuantel.mp. [mp=title, abstract, heading word, drug trade name, original title, device manufacturer, drug manufacturer, device trade name, keyword, floating subheading word, candidate term word] (9)

17 anthelminti*.mp. [mp=title, abstract, heading word, drug trade name, original title, device manufacturer, drug manufacturer, device trade name, keyword, floating subheading word, candidate term word] (17786)

18 antihelminti*.mp. [mp=title, abstract, heading word, drug trade name, original title, device manufacturer, drug manufacturer, device trade name, keyword, floating subheading word, candidate term word] (466)

19 chemoprevention.mp. [mp=title, abstract, heading word, drug trade name, original title, device manufacturer, drug manufacturer, device trade name, keyword, floating subheading word, candidate term word] (15536)

20 quimiopreven*.mp. [mp=title, abstract, heading word, drug trade name, original title, device manufacturer, drug manufacturer, device trade name, keyword, floating subheading word, candidate term word] (32)

21 chimioprevention.mp. [mp=title, abstract, heading word, drug trade name, original title, device manufacturer, drug manufacturer, device trade name, keyword, floating subheading word, candidate term word] (61)

22 mass drug administration.mp. [mp=title, abstract, heading word, drug trade name, original title, device manufacturer, drug manufacturer, device trade name, keyword, floating subheading word, candidate term word] (1857)

23 administration massive de medicaments.mp. [mp=title, abstract, heading word, drug trade name, original title, device manufacturer, drug manufacturer, device trade name, keyword, floating subheading word, candidate term word] (1)

24 8 or 9 or 10 or 11 or 12 or 13 or 14 or 15 or 16 or 17 or 18 or 19 or 20 or 21 or 22 or 23 (160795)

25 7 and 24 (1591)

26 editorial.pt. (572410)

27 (7 and 24) not 26 (1579)

***************************

**LILACS** – 31 July 2018

((mh:("taenia solium")) OR (mh:("taenia")) OR (tw:(taenia solium)) OR (tw:(taenia*)) OR (tw:(teniasis)) OR (tw:(teniase))) AND ((mh:("albendazole")) OR (mh:("niclosamide/")) OR (mh:("praziquantel/")) OR (mh:("anthelmintics")) OR (mh:("chemoprevention/")) OR (tw:(albendazol*)) OR (tw:(niclosamid*)) OR (tw:(praziquantel)) OR (tw:(prazicuantel)) OR (tw:(anthelminti*)) OR (tw:(antihelminti*)) OR (tw:(chemoprevention)) OR (tw:(quimiopreven*)) OR (tw:(chimioprevention)) OR (tw:(“mass drug administration”)) OR (tw:(“administración masiva de medicamentos”)) OR (tw:(“administration massive de medicaments”)) OR (tw:(“distribution massive de medicaments”)) OR (tw:(“administração massiva dos medicamentos”)) OR (tw:(deworm*))) AND (instance:"regional") AND ( db:("LILACS")) - 111

**SciELO** – 31 July 2018

((taenia solium) OR (taenia*) OR (teniasis) OR (teniase)) AND ((albendazol*) OR (niclosamid*) OR (praziquantel) OR (prazicuantel) OR (anthelminti*) OR (antihelminti*) OR (chemoprevention) OR (quimiopreven*) OR (chimioprevention) OR (mass drug administration) OR (administración masiva de medicamentos) OR (administration massive de medicaments) OR (distribution massive de medicaments) OR (administração massiva dos medicamentos) OR (deworm*)) – 32

**Cochrane Library (CENTRAL, Cochrane Database of Systematic Reviews, DARE, HTA, NHSEED)** – 31 July 2018

| ID | Search | Hits |
| --- | --- | --- |
| #1 | MeSH descriptor: [Taenia] explode all trees | 20 |
| #2 | MeSH descriptor: [Taenia solium] explode all trees | 8 |
| #3 | "taenia":ti,ab,kw (Word variations have been searched) | 50 |
| #4 | "Taenia solium":ti,ab,kw (Word variations have been searched) | 33 |
| #5 | teniasis:ti,ab,kw (Word variations have been searched) | 1 |
| #6 | taenia*:ti,ab,kw (Word variations have been searched) | 66 |
| #7 | teniase:ti,ab,kw (Word variations have been searched) | 0 |
| #8 | #1 or #2 or #3 or #4 or #5 or #6 or #7 | 69 |

Results per database:

CENTRAL – 67

CDSR – 1

DARE – 0

HTA – 0

NHSEED - 1

**Epistemonikos** – 31 July 2018

(title:(taenia) OR abstract:(taenia)) OR (title:(taenia*) OR abstract:(taenia*)) OR (title:(taenia solium) OR abstract:(taenia solium)) OR (title:(teniasis) OR abstract:(teniasis)) OR (title:(teniase) OR abstract:(teniase)) – 86

70 are classified as primary studies and 16 as systematic reviews

**CAB Abstracts** – 26 Sept 2018

((albendazol*) OR (niclosamid*) OR (praziquantel) OR (prazicuantel) OR (anthelmint*) OR (antihelmint*) OR (chemoprevention) OR (quimiopreven*) OR (chimioprevention) OR (mass drug administration) OR (administración masiva de medicamentos) OR (administration massive de medicaments) OR (distribution massive de medicaments) OR (administração massiva dos medicamentos) OR (deworm*)) AND (((teniasis) OR (teniase)) OR ((taenia) OR (taenia solium)))
