# Supplementary material for: Systematic review of the effectiveness of selected drugs for preventive chemotherapy for Taenia solium taeniasis
Source: PLoS Negl Trop Dis. 2020 Jan 16;14(1):e0007873. doi: 10.1371/journal.pntd.0007873 (PMC6964831; doi:10.1371/journal.pntd.0007873)
Supplement: S2 File — (DOCX) [file pntd.0007873.s003.docx]

**S2 File. Risk of bias assessment**

**Risk of bias for studies with a separate control group**

- Randomised trials
- Non-randomised trials
- Controlled before-after studies

1. **Random sequence generation (EPOC^^[[1]](#footnote-1)^^/Cochrane^^[[2]](#footnote-2)^^ tool)**

Score “Low risk” if a random component in the sequence generation process is described (e.g. Referring to a random number table). Score “High risk” when a nonrandom method is used (e.g. performed by date of admission). Non-randomised trials and controlled before-after studies should be scored “High risk”. Score “Unclear risk” if not specified in the paper.

*See Table 8.5.a and Table 8.5.d (Cochrane tool)*

1. **Allocation concealment (EPOC/Cochrane tool)**

Score “Low risk” if the unit of allocation was by institution, team or professional and allocation was performed on all units at the start of the study; or if the unit of allocation was by patient or episode of care and there was some form of centralised randomisation scheme, an on-site computer system or sealed opaque envelopes were used. Controlled before-after studies should be scored “High risk”. Score “Unclear risk” if not specified in the paper

*See Table 8.5.a and Table 8.5.d (Cochrane tool)*

1. **Blinding of participants and personnel (Cochrane tool)^^[[3]](#footnote-3)^^**

Describe all measures used, if any, to blind study participants and personnel from knowledge of which intervention a participant received. Provide any information relating to whether the intended blinding was effective.

*See Table 8.5.a and Table 8.5.d (Cochrane tool)*

1. **Blinding of outcome assessors (Knowledge of the allocated interventions adequately prevented during the study) (EPOC/Cochrane tool)**

Score “Low risk” if the authors state explicitly that the primary outcome variables were assessed blindly, or the outcomes are objective, e.g. length of hospital stay. Primary outcomes are those variables that correspond to the primary hypothesis or question as defined by the authors. Score “High risk” if the outcomes were not assessed blindly. Score “Unclear risk” if not specified in the paper.

*See Table 8.5.a and Table 8.5.d (Cochrane tool)*

1. **Baseline outcome measurements similar (EPOC)**

Score “Low risk” if performance or patient outcomes were measured prior to the intervention, and no important differences were present across study groups. In randomised trials, score “Low risk” if imbalanced but appropriate adjusted analysis was performed (e.g. Analysis of covariance). Score “High risk” if important differences were present and not adjusted for in analysis. If randomised trials have no baseline measure of outcome, score “Unclear risk”.

1. **Incomplete outcome data addressed (EPOC/Cochrane tool)**

Score “Low risk” if missing outcome measures were unlikely to bias the results (e.g. the proportion of missing data was similar in the pre- and post-intervention periods or the proportion of missing data was less than the effect size i.e. unlikely to overturn the study result). Score “High risk” if missing outcome data was likely to bias the results. Score “Unclear risk” if not specified in the paper (Do not assume 100% follow up unless stated explicitly).

*See Table 8.5.a and Table 8.5.d (Cochrane tool)*

1. **Selective outcome reporting (EPOC/Cochrane tool)**

Score “Low risk” if there is no evidence that outcomes were selectively reported (e.g. all relevant outcomes in the methods section are reported in the results section). Score “High risk” if some important outcomes are subsequently omitted from the results. Score “Unclear risk” if not specified in the paper.

*See Table 8.5.a and Table 8.5.d (Cochrane tool)*

1. **Other sources of bias (adapted from EPOC/Cochrane tool)**

Score “Low risk” if there is no evidence of other risk of biases. E.g. should consider if seasonality is an issue (i.e. if January to June comprises the pre-intervention period and July to December the post, could the “seasons’ have caused a spurious effect). Can include possible bias due to conflicts of interest.

*See Table 8.5.a and Table 8.5.d (Cochrane tool)*

**Risk of bias for studies with no control group:**

- Interrupted time series
- Before-after studies

1. **Intervention independent of other changes (EPOC)**

Score “Low risk” if there are compelling arguments that the intervention occurred independently of other changes over time and the outcome was not influenced by other confounding variables/historic events during study period. *If Events/variables identified, note what they are.* Score “High risk” if reported that intervention was not independent of other changes in time.

1. **Intervention unlikely to affect data collection (EPOC)**

Score “Low risk” if reported that intervention itself was unlikely to affect data collection (for example, sources and methods of data collection were the same before and after the intervention); Score “High risk” if the intervention itself was likely to affect data collection (for example, any change in source or method of data collection reported).

1. **Bias in selection of participants into the study (confounding) (adapted from ROBINS-I tool^^[[4]](#footnote-4)^^)**

Were baseline measurements conducted in a random sample of the eligible population?

1. **Blinding of outcome assessors (Knowledge of the allocated interventions adequately prevented during the study) (EPOC/Cochrane tool)**

Score “Low risk” if the authors state explicitly that the primary outcome variables were assessed blindly, or the outcomes are objective, e.g. length of hospital stay. Primary outcomes are those variables that correspond to the primary hypothesis or question as defined by the authors. Score “High risk” if the outcomes were not assessed blindly. Score “Unclear risk” if not specified in the paper.

*See Table 8.5.a and Table 8.5.d (Cochrane tool)*

1. **Incomplete outcome data addressed (EPOC/Cochrane tool)**

Score “Low risk” if missing outcome measures were unlikely to bias the results (e.g. the proportion of missing data was similar in the pre- and post-intervention periods or the proportion of missing data was less than the effect size i.e. unlikely to overturn the study result). Score “High risk” if missing outcome data was likely to bias the results. Score “Unclear risk” if not specified in the paper (Do not assume 100% follow up unless stated explicitly).

*See Table 8.5.a and Table 8.5.d (Cochrane tool)*

1. **Selective outcome reporting (EPOC/Cochrane tool)**

Score “Low risk” if there is no evidence that outcomes were selectively reported (e.g. all relevant outcomes in the methods section are reported in the results section). Score “High risk” if some important outcomes are subsequently omitted from the results. Score “Unclear risk” if not specified in the paper.

*See Table 8.5.a and Table 8.5.d (Cochrane tool)*

1. **Other risks of bias (adapted from EPOC/Cochrane tool)**

Score “Low risk” if there is no evidence of other risk of biases. E.g. should consider if seasonality is an issue (i.e. if January to June comprises the pre-intervention period and July to December the post, could the “seasons’ have caused a spurious effect). Can include possible bias due to conflicts of interest.

*See Table 8.5.a and Table 8.5.d (Cochrane tool)*

**Overall assessment:**

We scored each study for risk of bias as ‘low’ if all criteria were scored as ‘low’, ‘unclear’ if one or two criteria were scored as ‘unclear’ or ‘high’, and ‘high’ if more than two criteria were scored as ‘unclear’ or ‘high’ (Davey et al. 2017).

**References:**

Cochrane Effective Practice and Organisation of Care (EPOC). Suggested risk of bias criteria for EPOC reviews. EPOC Resources for review authors. Available at: [http://epoc.cochrane.org/resources/epoc-resources-review-authors. 2017](http://epoc.cochrane.org/resources/epoc-resources-review-authors.%202017)

Davey P, Marwick CA, Scott CL, Charani E, McNeil K, Brown E, Gould IM, Ramsay CR, Michie S. Interventions to improve antibiotic prescribing practices for hospital inpatients. Cochrane Database of Systematic Reviews 2017, Issue 2. Art. No.: CD003543.

Higgins JPT, Green S, editors. Cochrane Handbook for Systematic Reviews of Interventions Version 5.1.0 [updated March 2011]: The Cochrane Collaboration; 2011.

Sterne JA, Hernan MA, Reeves BC, Savovic J, Berkman ND, Viswanathan M, et al. ROBINS-I: a tool for assessing risk of bias in non-randomised studies of interventions. BMJ. 2016;355:i4919.

1. EPOC – refers to: Cochrane Effective Practice and Organisation of Care (EPOC). Suggested risk of bias criteria for EPOC reviews. EPOC Resources for review authors. Available at: [http://epoc.cochrane.org/resources/epoc-resources-review-authors. 2017](http://epoc.cochrane.org/resources/epoc-resources-review-authors.%202017) [↑](#footnote-ref-1)
2. Cochrane tool – refers to tool described in: Higgins JPT, Green S, editors. Cochrane Handbook for Systematic Reviews of Interventions Version 5.1.0 [updated March 2011]: The Cochrane Collaboration; 2011. [↑](#footnote-ref-2)
3. Included as part of: ‘Knowledge of the allocated interventions adequately prevented during the study’ in the EPOC tool. [↑](#footnote-ref-3)
4. Sterne JA, Hernan MA, Reeves BC, Savovic J, Berkman ND, Viswanathan M, et al. ROBINS-I: a tool for assessing risk of bias in non-randomised studies of interventions. BMJ. 2016;355:i4919. [↑](#footnote-ref-4)
