# Supplementary material for: Systematic review of the effectiveness of selected drugs for preventive chemotherapy for Taenia solium taeniasis
Source: PLoS Negl Trop Dis. 2020 Jan 16;14(1):e0007873. doi: 10.1371/journal.pntd.0007873 (PMC6964831; doi:10.1371/journal.pntd.0007873)
Supplement: S3 File — (DOCX) [file pntd.0007873.s004.docx]

# S3 File. List of excluded studies with reason for their exclusion, N=221

Note: complete references are listed after the table – pages 7-22

| Reference | Reason for exclusion |
| --- | --- |
| Abasov and Geibatov 1978 | Language - Russian |
| Abbasov, et al. 1979 | Language - Russian |
| Adonajlo, et al. 1969 | Language - Polish |
| Agrawal, 2017 | Participants - not T. solium |
| Ahmed 1983 | Participants - T. saginata |
| Alexander, et al. 2011 | Study type - cost analysis not an economic evaluation |
| Allan, et al. 2002 | Study type - narrative review |
| Alterio 1968 | Intervention - not NICL, PZQ or ALB |
| Amatoneto 1964 | Intervention - not NICL, PZQ or ALB |
| Anantaphruti, et al. 2010 | Outcomes; Study type - cross-sectional |
| Anantaraman 1974 | No abstract, No full text - cannot assess |
| Andre, et al. 1974 | No abstract, No full text - cannot assess |
| Anonymous 1975 | Study type - narrative review |
| Anonymous 1982 | No abstract, No full text - cannot assess |
| Apt 1983 | No abstract, No full text - cannot assess |
| Bagheri, 2004 | Study type - reporting of adverse events |
| Baranski 1977 | No full text - cannot assess |
| Baranski, et al. 1979 | No abstract, No full text - cannot assess |
| Baranski, et al. 1980 | No full text - cannot assess |
| Baranski, et al. 1984 | No full text - cannot assess |
| Barbieri, et al. 1993 | No full text - cannot assess |
| Barton Behravesh, et al. 2008 | Intervention - none given; Study type - cross-sectional |
| Batko and Kacka 1969 | Language - Polish |
| Beam, et al. 2018 | Intervention; Outcomes |
| Bekish and Bekish 2008 | Language - Russian |
| Botero Ramos and Ocampo 1982 | Abstract only, need full text |
| Botey 1984 | Participants - insufficient cases of T. solium |
| Bouree 1995 | No abstract, No full text - cannot assess |
| Bronshteĭn, et al. 1993 | Language - Russian |
| Bwibo and Pamba 1983 | Participants - not T. solium |
| Bwibo and Pamba 1984 | Participants - not T. solium |
| Camacho, et al. 1991 | Duplicate of Diaz Camacho et al. 1991 |
| Canedoacosta 1964 | Intervention - GS-1339 |
| Canto de Mena, et al. 1986 | No abstract, No full text - cannot assess |
| Carabin, et al. 2017 | Study type - narrative review |
| Carpio, et al. 2016 | Study type - letter |
| Carrada-Bravo 1987 | Study type - narrative review |
| Cavier and Notteghem 1968 | Study type |
| Chai 2013 | Study type - narrative review |
| Champetier de Ribes, et al. 2005 | Intervention - none given; Study type - cross-sectional |
| Chen, et al. 1995 | Language - Chinese |
| Chessed, et al. 2005 | Intervention - none given; Study type - cross-sectional |
| Chobanyan, et al. 1982 | Language - Russian |
| Chung, et al. 1991 | Participants - T. saginata |
| Chunge 1987 | No abstract, No full text - cannot assess |
| Copelman 1983 | No abstract, No full text - cannot assess |
| Coulaud, et al. 1982 | Participants - T. saginata |
| Cruz 1991 | Participants - T. saginata; Study type - narrative review |
| Cruz 1991 | Participants - T. saginata; Study type - narrative review |
| Damen, et al. 2010 | Intervention - mebendazole |
| Davis, 1979 | Participants - not T. solium; Study type - narrative review |
| Dettori, et al. 1994 | Intervention - none given; Language - Italian |
| Devleesschauwer, et al. 2012 | Intervention - none given; Study type - cross-sectional |
| Diaz, et al. 1992 | Intervention - none given; Study type - cross-sectional |
| Dickson 1983 | No full text - cannot assess |
| Dickson 1984 | No full text - cannot assess |
| Donckaster, et al. 1961 | No full text - cannot assess |
| Draghici, et al. 1974 | Language - Rumanian |
| Du, et al. 2012 | Language - Chinese (otherwise meets criteria) |
| Dufek and Kalivoda 1969 | Participants- T. saginata |
| Dzhumaev 1996 | Language - Russian |
| Espejo 1977 | No abstract, No full text - cannot assess |
| Esteves, et al. 2005 | Intervention - none given; Study type - cross-sectional |
| Fan, et al. 1982 | No full text - cannot assess |
| Fan, et al. 1986 | No full text - cannot assess |
| Fernandez Ortega 1982 | No full text - cannot assess |
| Flisser 2002 | Study type - narrative review |
| Flisser, et al. 1995 | Participants - not T. solium |
| Foba-Pagou, et al. 1980 | No abstract, No full text - cannot assess |
| Fontes, et al. 2003 | Intervention - can't attribute the results to one of the included drugs |
| Frohberg 1984 | Study type - toxicology |
| Frohberg 1989 | Study type - narrative review |
| Fu, et al. 1988 | Participants - not T. solium; Study type - narrative review |
| Gabriel, et al. 2017 | Study type - narrative review |
| Gabrielli, 2011 | Study type - narrative review |
| Gamboa, 2017 | Abstract only, need full text to be able to include |
| Garcia, 2010 | Study type - narrative review |
| Garcia, et al. 2006 | Outcomes - in pigs only |
| Garcia, et al. 2016 | Insufficient information on methods and results (in humans) of MDA to include |
| Garrido Lecona 1975 | Participants - not T. solium |
| Geerts 1994 | Study type - narrative review |
| Geisenhainer 1968 | Language - German |
| Gilles 1976 | Study type - narrative review |
| Gilman, et al. 2012 | Study type - narrative review |
| Glisic, et al. 1972 | Language - Serbo-Croatian |
| Goldman, 2011 | Participants - not T. solium |
| Goldsmid and Fleming 1977 | Intervention - none given; Study type - cross-sectional |
| Gonzalez, et al. 2003 | Study type - narrative review |
| Groll 1977 | No abstract, No full text - cannot assess |
| Groll 1983 | No full text - email author |
| Gupte 1977 | Study type - narrative review |
| Hamid, et al. 2005 | Study type - narrative review |
| Hamidu, et al. 2014 | No full text - cannot assess |
| Haznedaroğlu, et al. 1994 | Participants - not T. solium; Language - Turkish |
| Homeida and Musa 1984 | Participants - T. saginata |
| Hong 2018 | Participants - not T. solium |
| Hong, et al. 1983 | Language - Korean |
| Hotez 2014 | Study type - narrative review |
| Howarth, et al. 1988 | Participants - not T. solium |
| Huggins 1981 | Intervention - flubendazole |
| Huggins 1982 | No full text - cannot assess |
| Huggins 1985 | Intervention - flubendazole |
| Huggins 1987 | No full text - cannot assess |
| Huggins, et al. 1993 | No abstract, No full text - cannot assess |
| Ibrahim 1983 | No abstract, No full text - cannot assess |
| Imbert and Moulin 2010 | Study type - narrative review |
| Ioli, et al. 1987 | Participants - not T. solium |
| Jeon, et al. 2013 | Intervention - none given |
| Joo 1984 | Intervention - none given; Study type - cross-sectional |
| Kabatereine, 2003 | Participants - not T. solium |
| Karaman, et al. 2006 | Language - Turkish |
| Karnaukhov 1982 | Study type - narrative review |
| Keiser, 2008 | Participants - not T. solium |
| Kia, et al. 2005 | Participants - T. saginata; Study type - cross-sectional |
| Kihara 1989 | Language - Japanese |
| Kimani, 2018 | Participants - not T. solium |
| Kociecka 1987 | No abstract, No full text - cannot assess |
| Komma and Santos 1972 | Study type - cross-sectional |
| Koul, et al. 2004 | Study type - letter |
| Kurup 2010 | Participants - not T.solium endemic (or suspected endemic) country. Taenia spp. prevalence at baseline 0.2% (2/897). Intervention – not clear which drug was used to treat the 2 cases |
| Kurup and Hunjan 2010 | Participants - not T.solium endemic (or suspected endemic) country |
| Kuzacute˜micki, et al. 1985 | Language - Polish |
| Lara-Aguilera, et al. 1990 | Participants - not T. solium |
| Laranjo-Gonzalez, et al. 2017 | Intervention - none given |
| Latham, et al. 1983 | Study type - cross-sectional |
| Lazar and Cristian-Davidescu 2004 | Language - Rumanian; Intervention - purgatives |
| Levay and Vilimszky 1975 | Language - Hungarian |
| Li, et al. 2013 | Intervention - pumpkin seeds and areca; Language - Chinese |
| Lightowlers, et al. 2016 | Study type - narrative review of diagnostic tests |
| Long, et al. 2014 | Language - Chinese (otherwise meets criteria) |
| Louzada 1979 | No abstract, No full text - cannot assess |
| Louzada, et al. 1978 | No abstract, No full text - cannot assess |
| Louzada, et al. 1979 | No abstract, No full text - cannot assess |
| Louzada, et al. 1987 | No full text - cannot assess |
| Madinga, et al. 2017 | Intervention - none given; Study type - cross-sectional |
| Magdieva 1992 | Language - Russian |
| Margono, et al. 2006 | Study type - narrative review |
| Martin del Barco, et al. 2009 | Study type - cross-sectional |
| Martinez Baez, et al. 1971 | Study type |
| Marty, et al. 1986 | No full text - cannot assess |
| McCleery, et al. 2015 | Study type - cross-sectional |
| Mejia, et al. 2015 | Study type - narrative review |
| Misra, 1985 | Participants - not T. solium |
| Mohammed, 2008 | Participants - not T. solium |
| Montresor and Palmer 2006 | Study type - narrative review |
| Most, et al. 1971 | Participants - not T. solium |
| Mwanjali, et al. 2013 | Intervention - none given; Study type - cross-sectional |
| Mwape, et al. 2012 | Intervention - none given; Study type - cross-sectional |
| Nct 2011 | Intervention - community-led total sanitation; Outcomes - cysticercosis |
| Nct 2015 (Gabriel & Mwape) | Study in progress, need results to assess to be able to include |
| Neghina, et al. 2010 | Study type - retrospective chart review |
| Njomo, 2010 | Participants - not T. solium |
| Nwosu 1982 | No abstract, No full text - cannot assess |
| Nwosu 1982 | No full text - cannot assess |
| O'Neal, et al. 2017 | No full text – need results to be able to include |
| Ofori-Adjei, 2008 | Study type - safety review |
| Ohnishi, et al. 2013 | Participants - T. asiatica |
| Okello and Thomas 2017 | Study type - narrative review |
| Okelo 1984 | No full text - cannot assess |
| Olveda, et al. 1983 | No abstract, No full text - cannot assess |
| Onkanga, 2016 | Participants - not T. solium |
| Patwari, et al. 1980 | No abstract, No full text - cannot assess |
| Pawlowski 1987 | Study type - narrative review |
| Pawlowski 1990 | Study type - narrative review |
| Pawłowski 1990 | Study type - narrative review |
| Pawlowski 1991 | Study type - narrative review |
| Pawłowski 1991 | Study type - narrative review |
| Pawlowski 2006 | Study type - narrative review |
| Pawlowski, et al. 2005 | Study type - narrative review |
| Pearson and Guerrant 1983 | Study type - narrative review |
| Pearson and Hewlett 1985 | Study type - narrative review |
| Pene, et al. 1982 | Participants - T. saginata |
| Penrith, 2009 | Study type - conference report |
| Perera, et al. 1970 | Participants - T. saginata (1 had T. solium) |
| Pierattelli 2001 | Language - Italian |
| Plotnikov, et al. 1972 | Intervention - pumpkin seeds; Language - Russian |
| Prasad, et al. 2007 | Study type - cross-sectional |
| Radoev 1974 | Language - Bulgarian |
| Radomyos, et al. 1984 | Participants - flukes |
| Rajshekhar 2004 | Study type - editorial |
| Ramos-Zúñiga, et al. 2013 | Study type - report of 5 cases with NCC taking albendazole |
| Raso, 2004 | Participants - not T. solium |
| Richards and Schantz 1985 | Study type - narrative review/debate |
| Rim, et al. 1978 | Language - Korean |
| Rodriguez-Canul, et al. 1999 | Study type - cross-sectional |
| Rossignol 1981 | No abstract, No full text - cannot assess |
| Rossignol and Maisonneuve 1984 | Study type - narrative review |
| Rossignol, 1983 | Participants - not T. solium |
| Sanchez, et al. 1997 | Intervention - none given; Study type - cross-sectional |
| Sanchez, et al. 1998 | Intervention - none given; Study type - cross-sectional |
| Santana, et al. 2015 | Intervention - none given; Study type - cross-sectional |
| Sarti and Rajshekhar 2003 | Study type - narrative review |
| Sarti, et al. 1988 | Abstract only, need full text |
| Sarti, et al. 1994 | Study type - cross-sectional |
| Sato, et al. 2018 | Study type - cross-sectional |
| Shafei 1965 | No full text - cannot assess |
| Singh, et al. 2004 | No full text - cannot assess |
| Sircar, et al. 2018 | Participants - not T. solium |
| Sobota 1969 | Language - Slovakian |
| Soh, et al. 1976 | Language - Korean |
| Soh, et al. 1976 | Study type – no baseline measures |
| Stahel 1977 | Language - German |
| Steinmann, et al. 2014 | Duplicate of Steinmann et al. 2015 |
| Suroso, et al. 2006 | Study type - narrative review |
| Tanowitz, et al. 1993 | Study type - narrative review |
| Tanowitz, et al. 2001 | Study type - narrative review |
| Thi Thi, et al. 2015 | Participants - not T. solium endemic (or suspected endemic) country. Prevalence of Taenia spp. 0.1% (1/1443) at baseline and 0.3% (3/1084) at follow-up. |
| Tianzuan, et al. 1991 | Language - Chinese |
| Todorov 1973 | Language - Bulgarian |
| Torres, et al. 2001 | Study type - case series |
| Van De, et al. 2014 | Study type - narrative review |
| Vidal, et al. 2016 | No full text – need results to be able to include |
| Vitta, et al. 2012 | Study type - cross-sectional |
| Wandra, et al. 2006 | Study type - narrative review |
| Wandra, et al. 2011 | Study type - narrative review |
| Watts, et al. 2014 | Study type - cross-sectional |
| Wilson, et al. 1987 | Study type - cross-sectional |
| Xiao, et al. 2013 | Intervention - tribendimidine; Study type - narrative review |
| Yap, et al. 2014 | Outcome - physical fitness |
| Zhang 1985 | Language - Chinese |
| Zhang 2003 | Language - Chinese |

## References to excluded studies:

1. Abasov KD, Geibatov AD. Treatment of patients with taeniasis with trichlosal tablets in out-patient clinics. Sbornik Nauchnykh Trudov Nauchno-Issledovatel'skogo Instituta Meditsinskoi Parazitologii i Tropicheskoi Meditsiny, Baku (Nauchnye osnovy snizheniya i likvidatsii parazitarnykh boleznei v Azerbaidzhanskoi SSR). 1978(10):43-5.

2. Abbasov KD, Chobanov RE, Khalilov AS, Geibatov AD. Epidemiological features of helminthiases and the organization of effective control measures in the Kubinskii region. Baku: Ministerstvo Zdravookhraneniya Azerbaidzhanskoi SSR.; 1979. p. 15-30.

3. Adonajlo A, Bonczak J, Ganearz Z. Epidemiologic and epizootiologic situation of taeniasis and cysticercosis in Poland in the years 1965-1967. [Polish]. PrzeglEpidem. 1969;23(4):473-6.

4. Agrawal P, Srivastava B, Bhardwaj R, Gaur S. Adverse events of albendazole due to mass drug administration. International Journal of Basic & Clinical Pharmacology. 2017;6(7):1674-7.

5. Ahmed MEK. Albendazole in the Sudan (open study of albendazole in the treatment of intestinal helminthiasis). London; Royal Society of Medicine; Academic Press. New York, USA: Grune & Stratton; 1983. p. 39-43.

6. Alexander A, John K, Jayaraman T, Oommen A, Venkata RM, Dorny P, et al. Economic implications of three strategies for the control of taeniasis (Structured abstract). Tropical Medicine and International Health [Internet]. 2011; 16(11):[1410-6 pp.]. Available from: <http://cochranelibrary-wiley.com/o/cochrane/cleed/articles/NHSEED-22012039160/frame.html> https://onlinelibrary.wiley.com/doi/pdf/10.1111/j.1365-3156.2011.02850.x.

7. Allan JC, Craig PS, Pawlowski ZS. Control of Taenia solium with emphasis on treatment of taeniasis. 2002. In: Taenia Solium Cysticercosis: From Basic to Clinical Science [Internet]. Wallingford: CABI Publishing; [411-20]. Available from: http://www.cabi.org/cabebooks/ebook/20023159884.

8. Alterio DL. [Treatment of teniasis with cholorhydroxysalicylamide and diphenthane]. Revista do Hospital das Clinicas. 1968;23(5):259-61.

9. Amatoneto V. [TREATMENT WITH A SALICYLAMIDE DERIVATIVE OF TAENIA SAGINATA AND TAENIA SOLIUM INFESTATIONS]. Revista do Instituto de Medicina Tropical de Sao Paulo. 1964;6:297-9.

10. Anantaphruti MT, Okamoto M, Yoonuan T, Saguankiat S, Kusolsuk T, Sato M, et al. Molecular and serological survey on taeniasis and cysticercosis in Kanchanaburi Province, Thailand. Parasitology international. 2010;59(3):326-30.

11. Anantaraman M. The prevalence and transmission of human taeniasis in India. Vienna: FACTA Publication.; 1974. p. 394-5.

12. Andre LJ, Sirol J, Delprat J. Treatment of intestinal helminthiases. [French]. Medecine et Armees. 1974;2(5):465-8.

13. Anonymous. Chermotherapy for infection with worms and flukes. Drug and Therapeutics Bulletin. 1975;13(17):65-8.

14. Anonymous. 'Albendazole in the treatment of tapeworm infestation'. American Journal of Gastroenterology. 1982;77(10):19.

15. Apt W. Treatment of intestinal helminthiasis. Parasitología al día. 1983;7(3):83-4.

16. Bagheri H, Simiand E, Montastruc J-L, Magnaval J-F. Adverse Drug Reactions to Anthelmintics. Annals of Pharmacotherapy. 2004;38(3):383-8.

17. Baranski MC. Treatment of human taeniasis and hymenolepiasis with praziquantel (Embay 8440). [Spanish]. Boletin chileno de parasitologia. 1977;32(1-2):37-9.

18. Baranski MC, Gomes NR, De Godoy OF. Treatment of taeniasis and of Hymenolepis nana with a single oral dose of praziquantel. Study of the efficacy tolerance and safety. [Portuguese]. Revista do Instituto de Medicina Tropical de Sao Paulo. 1980;22(2):82-8.

19. Baranski MC, Gomes NR, Godoy FO, Silva FA, Kotaka IP, Cunha CAM, et al. The treatment of intestinal taeniasis and of hymenolepiasis nana with single oral doses of praziquantel (Embay 8440). Study of efficiency, tolerance and safety. . 1979. p. [201].

20. Baranski MC, Gomes NR, Godoy OFd, Silva AFd, Kotaka PI, Giovannoni M, et al. Treatment of taeniasis and Hymenolepis nana with a single oral dose of praziquantel. Study of therapeutic efficacy, tolerance and safety. Materia Medica Polona. 1984;16(2/4):129-33.

21. Barbieri D, Rodrigues M, Romaldini C. Albendazole in the treatment of intestinal helminthiasis in paediatric patients. Revista Brasileira de Medicina. 1993;50(4):362-6.

22. Barton Behravesh C, Mayberry LF, Bristol JR, Cardenas VM, Mena KD, Martinez-Ocana J, et al. Population-based survey of taeniasis along the United States-Mexico border. Annals of tropical medicine and parasitology. 2008;102(4):325-33.

23. Batko B, Kacka I. Coexistence of taeniasis with other parasitic infections. Wiadomosci Lekarskie. 1969;22(18):1663-5.

24. Beam M, Spencer A, Fernandez L, Atto R, Muro C, Vilchez P, et al. Barriers to Participation in a Community-Based Program to Control Transmission of Taenia solium in Peru. The American journal of tropical medicine and hygiene. 2018;98(6):1748-54.

25. Bekish VY, Bekish OYL. New approaches to medical treatment of human taenidosis. St. Petersburg: Russian Society of Parasitologists - Russian Academy of Sciences; 2008. p. 51-4.

26. Botero Ramos D, Ocampo NE. Tratamiento de Teniasis y de Himenolepiasis con Praziquantel. Colomb Med (Cali). 1982;13(4):131-4.

27. Botey MA. Clinical development of albendazole in Latin America. London: Royal Society of Medicine; 1984. p. 115-9.

28. Bouree P. Taeniasis. [French]. Revue du Praticien - Medecine Generale. 1995;9(308):11-2.

29. Bronshteĭn AM, Mel'nikova LI, Firsova RA, Legon'kov YA. Analysis of the results of clinical trials of analogues of praziquantel against intestinal cestode and trematode infections. 1. Treatment of intestinal cestode infections (diphyllobothriasis, taeniasis, hymenolepiasis). Meditsinskaya Parazitologiya i Parazitarnye Bolezni. 1993(No. 3):27-9.

30. Bwibo NO, Pamba HO. Double-blind comparative study of albendazole and placebo in the treatment of intestinal helminths. London; Royal Society of Medicine; Academic Press. New York, USA: Grune & Stratton; 1983. p. 49-59.

31. Bwibo NO, Pamba HO. Double-blind comparative study of albendazole and placebo in the treatment of intestinal helminths. London: Royal Society of Medicine; 1984. p. 47-53.

32. Camacho SPD, Ruiz AC, Peraza VS, Ramos MLZ, Medina MF, Lozano R, et al. Epidemiological study and control of Taenia solium infections with praziquantel in a rural village of Mexico. American Journal of Tropical Medicine and Hygiene. 1991;45(4):522-31.

33. Canedoacosta J. [CLINICAL EVALUATION OF GS-1339 IN THE TREATMENT OF TAENIA INFECTIONS]. Revista de investigacion. 1964;4:105-10.

34. Canto de Mena B, Rosario S, Hurst J. Terapia combinada en el manejo de la teniasis intestinal. Rev Med Dominic. 1986;47(2):11-3.

35. Carabin H, Winkler AS, Dorny P. Taenia solium cysticercosis and taeniosis: Achievements from the past 10 years and the way forward. PLoS neglected tropical diseases. 2017;11 (4) (no pagination)(e0005478).

36. Carpio A, Fleury A, Parkhouse M. Elimination of taenia solium transmission in Peru. New England Journal of Medicine. 2016;375(12):1196.

37. Carrada-Bravo T. The epidemiology of taeniasis-cysticercosis. [Spanish]. Boletin medico del Hospital Infantil de Mexico. 1987;44(7):427-34.

38. Cavier R, Notteghem MJ. A pharmacologie trial of some anthelminthics effective against intestinal cestodes of man. [French]. Annpharm. 1968;Franc 26(9-10):603-6.

39. Chai JY. Human taeniasis in the Republic of Korea: Hidden or gone? Korean Journal of Parasitology. 2013;51(1):9-17.

40. Champetier de Ribes G, Fline M, Desormeaux AM, Eyma E, Montagut P, Champagne C, et al. [Intestinal helminthiasis in school children in Haiti in 2002]. Bulletin de la Societe de pathologie exotique (1990). 2005;98(2):127-32.

41. Chen XY, Zhao YJ, Lian ZQ, Fang C. Taeniasis treated with albendazole and praziquantel. The journal of dalian medical cllege. 1995;4(1):28‐9.

42. Chessed G, Kwalagbe B, Furo NA. Intestinal helminthiases among school children in Gyawana District, Adamawa State, Nigeria. Global Journal of Pure and Applied Sciences. 2005;11(2):193-6.

43. Chobanyan AG, Zakaryan GG, Arutyunian RA, Balasanyan MA. The epidemiology of cysticercosis in cattle and pigs and the prevalence of taeniasis in the population of Armenia. Zoologicheskii Sbornik, Akademiya Nauk Armyanskoi SSR, Institut Zoologii (Fauna parazitov zhivotnykh i vyzyvaemye imi zabolevaniya). 1982(18):154-7.

44. Chung WC, Fan PC, Lin CY, Wu CC. Poor efficacy of albendazole for the treatment of human taeniasis. International journal for parasitology. 1991;21(2):269‐70.

45. Chunge CN. Praziquantel for the treatment of tapeworms in Kenya. East African medical journal. 1987;64(10):672-4.

46. Copelman H. Praziquantel, clinical trial on taeniasis. [Portuguese]. Folha Medica. 1983;86(1-2):87-9.

47. Coulaud JP, Duchatelle C, Rouvillois A, Deluol AM. Zentel in the treatment of intestinal helminthiases in Niger, Gabon and Paris. Medecine d'Afrique Noire. 1982;29(Numero Special):41-2.

48. Cruz AC. Treatment of human taeniasis in the Philippines: a review. The Southeast Asian journal of tropical medicine and public health. 1991;22 Suppl:271-4.

49. Cruz AC. Treatment of human taeniasis in the Philippines: a review. Bangkok: SEAMEO Regional Tropical Medicine & Public Health Project; 1991. p. 271-4.

50. Damen JG, Lar P, Mershak P, Mbaawuga EM, Nyary BW. A comparative study on the prevalence of intestinal helminthes in dewormed and non-dewormed students in a rural area of north-central Nigeria. Laboratory Medicine. 2010;41(10):585-9.

51. Davis A, Wegner DH. Multicentre trials of praziquantel in human schistosomiasis: design and techniques. Bulletin of the World Health Organization. 1979;57(5):767-71.

52. Dettori G, Viani I, Manini M, Calderaro A, Chezzi C. Results of the parasitological investigations carried out in the first eighteen months of activity (May 1992-December 1993) at the Institute of Microbiology, University of Parma. Igiene Moderna. 1994;101(1):71-99.

53. Devleesschauwer B, Aryal A, Joshi DD, Rijal S, Sherchand JB, Praet N, et al. Epidemiology of taenia solium in Nepal: Is it influenced by the social characteristics of the population and the presence of Taenia asiatica? Tropical Medicine and International Health. 2012;17(8):1019-22.

54. Diaz F, Garcia HH, Gilman RH, Gonzales AE, Castro M, Tsang VC, et al. Epidemiology of taeniasis and cysticercosis in a Peruvian village. The Cysticercosis Working Group in Peru. American journal of epidemiology. 1992;135(8):875-82.

55. Dickson B. Summary of clinical trials in the Middle East and Africa. London; Royal Society of Medicine; Academic Press. New York, USA: Grune & Stratton; 1983. p. 31-8.

56. Dickson B. Summary of albendazole clinical trials in the Middle East and Africa. London: Royal Society of Medicine; 1984. p. 79-84.

57. Donckaster R, Donoso F, Atias A, Faiguenbaum J, Jarpa A. [Trial therapy of teniasis with a derivative of salicylamide (Yomesan Baver). Preliminary note]. Boletin chileno de parasitologia. 1961;16:4-6.

58. Draghici O, Draghici G, Vasadi T. [Taeniasis]. Bacteriologia, virusologia, parazitologia, epidemiologia. 1974;19(2):159-64.

59. Du Z, Jiang J, Zhou H, Wang X, Chen R, Wang L, et al. Efficacy of different doses of albendazole and mebendazole against common soil-transmitted Helminths and Taenia spp.: a randomized controlled trial. Zhongguo Bingyuan Shengwuxue Zazhi / Journal of Pathogen Biology. 2012;7(5):360-2, 6.

60. Dufek M, Kalivoda R. Experience with modern treatment of taenlasis. Review of Czechoslovak Medicine. 1969;15(1):9-13.

61. Dzhumaev MD. Organization of taeniasis control in the Bukhara region. Meditsinskaya Parazitologiya i Parazitarnye Bolezni. 1996(3):55-6.

62. Espejo H. [Treatment of infections by Hymenolepis nana, Taenia saginata, Taenia solium and Diphyllobothrium pacificum with praziquantel (Embay 8440) (author's transl)]. Boletin chileno de parasitologia. 1977;32(1-2):39-40.

63. Esteves FM, Silva-Vergara ML, Carvalho AC. [Epidemiologic survey of teniasis in Health and Family Program in Uberaba, MG]. Revista da Sociedade Brasileira de Medicina Tropical. 2005;38(6):530-1.

64. Fan PC, Chung WC, Chan CH, Chen YA, Cheng FY, Hsu MC. Studies on taeniasis in Taiwan. V. Field trial on evaluation of therapeutic efficacy of mebendazole and praziquantel against taeniasis. Southeast asian j trop med pub hlth. 1986;17(1):82‐90.

65. Fan PC, Chung WC, Chan CH, Lee KM, Wang CC, Wu CC. A pilot control study of common intestinal parasites and head louse among aboriginal children in Taiwan, ROC. Part 1. Prevalence and chemotherapy of parasitic infections among aboriginal children in Nan-Ao and Ta-Tung Districts, Ilan County, northeastern Taiwan. National Science Council Monthly. 1982;10(9):797-8.

66. Fernandez Ortega F. Single dose treatment of albendazole for intestinal parasites in children. Report of 100 cases. Investigacion Medica Internacional. 1982;9(2):124-6.

67. Flisser A. Risk factors and control measures for taeniosis/cysticercosis. In: Craig P, Pawlowski Z, editors. Proceedings of the NATO Advanced Research Workshop on cestode zoonoses: echinococcosis and cysticercosis: an emergent and global problem, Poznan, Poland, 10-13 September 2000. Amsterdam: IOS Press; 2002. p. 335-42.

68. Flisser A, Sarti E, Sarti R, Schantz PM, Valencia S. Effect of praziquantel on protozoan parasites [2]. Lancet (London, England). 1995;345(8945):316-7.

69. Foba-Pagou R, Kegoum E, Same-Ekobo A. An epidemiological study of intestinal helminthiasis (Ascariasis, Necatoriasis, Taeniasis, Bilharziasis) in the town of Maroua (North Cameroon). [French]. Bulletin de la Societe de Pathologie Exotique et de ses Filiales. 1980;73(2):171-8.

70. Fontes G, Oliveira KKL, Oliveira AKL, Rocha EMMd. Influence of specific treatment of intestinal parasites and schistosomiasis on prevalence in students in Barra de Santo Antônio, AL. Revista da Sociedade Brasileira de Medicina Tropical. 2003;36(5):625-8.

71. Frohberg H. Results of toxicological studies on praziquantel. Arzneimittel-Forschung. 1984;34(9b):1137-44.

72. Frohberg H. The toxicological profile of praziquantel in comparison to other anthelminthic drugs. Acta Leidensia. 1989;57(2):201-15.

73. Fu S, Xiao SH, Catto BA. Clinical use of praziquantel in China. Parasitology Today. 1988;4(11):312-5.

74. Gabriel S, Dorny P, Mwape KE, Trevisan C, Braae UC, Magnussen P, et al. Control of Taenia solium taeniasis/cysticercosis: The best way forward for sub-Saharan Africa? Acta tropica. 2017;165:252-60.

75. Gabrielli AF, Montresor A, Chitsulo L, Engels D, Savioli L. Preventive chemotherapy in human helminthiasis: theoretical and operational aspects. Transactions of the Royal Society of Tropical Medicine and Hygiene. 2011;105(12):683-93.

76. Gamboa R, Vilchez P, Moyano LM, Muro C, Benavides V, O’Neil SE, et al. Efficacy and adverse events of niclosamide in a large scale cysticercosis elimination demonstration program on the North Coast of Peru. American Journal of Tropical Medicine and Hygiene. 2017;95(5 Suppl):140.

77. Garcia HH, Gonzalez AE, Gilman RH, Moulton LH, Verastegui M, Rodriguez S, et al. Combined human and porcine mass chemotherapy for the control of T. solium. The American journal of tropical medicine and hygiene. 2006;74(5):850-5.

78. Garcia HH, Gonzalez AE, Rodriguez S, Gonzalvez G, Llanos-Zavalaga F, Tsang VC, et al. [Epidemiology and control of cysticercosis in Peru]. Revista peruana de medicina experimental y salud publica. 2010;27(4):592-7.

79. Garcia HH, Gonzalez AE, Tsang VC, O'Neal SE, Llanos-Zavalaga F, Gonzalvez G, et al. Elimination of Taenia solium Transmission in Northern Peru. The New England journal of medicine. 2016;374(24):2335-44.

80. Garrido Lecona M. Collective intestinal desparasitization with a new atoxic polyvalent antiparasitic preparation. [Spanish]. Medicina. 1975;55(1199):255-9.

81. Geerts S. The efficacy of praziquantel for the treatment of cestode and metacestode infections. International Journal of Antimicrobial Agents. 1994;4(4):321-4.

82. Geisenhainer G. [Results of the treatment in taenia infection]. Zeitschrift fur arztliche Fortbildung. 1968;62(23):1266-7.

83. Gilles HM. Diseases of the alimentary system; treatment of intestinal worms. British Medical Journal. 1976;2(6047):1314-6.

84. Gilman RH, Gonzalez AE, Llanos-Zavalaga F, Tsang VCW, Garcia HH. Prevention and control of Taenia solium taeniasis/cysticercosis in Peru. Pathogens and global health. 2012;106(5):312-8.

85. Glisic L, Sretenovic M, Simic P. Our experience in the treatment of Taenia infection in humans. Acta Parasitologica Iugoslavica. 1972;3(1):27-30.

86. Goldman AS, Brady MA, Direny A, Desir L, Oscard R, Vely JF, et al. Costs of integrated mass drug administration for neglected tropical diseases in Haiti. The American journal of tropical medicine and hygiene. 2011;85(5):826-33.

87. Goldsmid JM, Fleming F. The tapeworm infections of children in Rhodesia. Central African Journal of Medicine. 1977;23(1):7-10.

88. Gonzalez AE, Garcia HH, Gilman RH, Tsang VCW. Control of Taenia solium. Acta tropica. 2003;87(1):103-9.

89. Groll E. General scope of treatment with praziquantel (Embay 8440) in human cestode infections. [Spanish]. Boletin chileno de parasitologia. 1977;32(1-2):27-31.

90. Groll E. Taeniasis - a disease which should be treated. Prague: Academia, Praha; 1983. p. 288-92.

91. Gupte S. Current trends in drug therapy of intestinal parasites. Indian journal of pediatrics. 1977;44(354):195-8.

92. Hamid A, Margono SS, Wandra T, Ito A. Treatment of taeniasis and cysticercosis with praziquantel and albendazole. Medical Journal of Indonesia. 2005;14(4):253-7.

93. Hamidu BB, Tettevi EJ, Larbi JA, Osei-Atweneboana MY. The efficacy of albendazole and levamisole drug combination in individuals with reduced efficacy for single-dose albendazole treatment against hookworm infections. American Journal of Tropical Medicine and Hygiene. 2014;1):152.

94. Haznedaroğlu T, Tanyüksel M, Gün H. Continuation of nocturnal enuresis following anti-parasitic treatment. Türk Hijyen ve Deneysel Biyoloji Dergisi. 1994;51(1):35-9.

95. Homeida M, Musa ARM. Open study of albendazole in the treatment of intestinal helminthiasis in the Sudan (preliminary report). London: Royal Society of Medicine; 1984. p. 75-8.

96. Hong ST. Albendazole and Praziquantel: Review and Safety Monitoring in Korea. Infect Chemother. 2018;50(1):1-10.

97. Hong ST, Hong SJ, Lee SH. A study on the intestinal helminths of the patients in a leprosarium in Korea. [Korean]. Korean Journal of Parasitology. 1983;21(1):102-4.

98. Hotez PJ. Neglected Parasitic Infections and Poverty in the United States. PLoS neglected tropical diseases. 2014;8(9).

99. Howarth SE, Wilson JM, Ranaivoson E, Crook SE, Denning AM, Hutchings MS. Worms, wells and water in Western Madagascar. Journal of Tropical Medicine and Hygiene. 1988;91(5):255-64.

100. Huggins D. Clinical trial of flubendazole in teniasis. [Portuguese]. Anais do Instituto de Higiene e Medicina Tropical. 1981;7(1-4):85-6.

101. Huggins D. Two news drugs for the treatment of intestinal parasitosis. [Portuguese]. Revista Brasileira de Medicina. 1982;39(9):565-8.

102. Huggins D. Ensaio clínico com flubendazol no tratamento das teníases. Bol Cient Soc Flumin Med Cir. 1985;17(1):23-4.

103. Huggins D. Teníases. Med HUPE-UERJ. 1987;6(3):207-11.

104. Huggins D, Hinrichesen SL, Arruda CS, Medeiros LB, Fragoso V, Oliveira ERd. Helmintiase na infância. Pediatr mod. 1993;29(4):529-30, 32-4, 36-8 passim.

105. Ibrahim AMA. Intestinal protozoal and helminthic infections in a university community. East African medical journal. 1983;60(1):64-7.

106. Imbert P, Moulin F. Anthelmintic drugs in children. [French]. Archives de Pediatrie. 2010;17(6):840-1.

107. Ioli A, Mento G, Leonaldi R, Vasi A, Verzera A, Lento FG, et al. Albendazole in the treatment of intestinal helminthiasis: study on 140 patients. Rivista di Parassitologia. 1987(3):291-6.

108. Jeon HK, Yong TS, Sohn WM, Chai JY, Min DY, Yun CH, et al. Current status of human taeniasis in Lao People's Democratic Republic. Korean Journal of Parasitology. 2013;51(2):259-63.

109. Joo CY. Recent patterns of intestinal helminth infections among the residents of Taegu City, Korea. Korean Journal of Parasitology. 1984;22(1):109-15.

110. Kabatereine NB, Kemijumbi J, Ouma JH, Sturrock RF, Butterworth AE, Madsen H, et al. Efficacy and side effects of praziquantel treatment in a highly endemic Schistosoma mansoni focus at Lake Albert, Uganda. Transactions of the Royal Society of Tropical Medicine and Hygiene. 2003;97(5):599-603.

111. Karaman Ü, Atambay M, Aycan Ö, Yoloğlu S, Daldal N. Incidence of intestinal parasites in municipal sanitary workers in Malatya. Türkiye Parazitoloji Dergisi. 2006;30(3):181-3.

112. Karnaukhov VK. Clinical aspects and treatment of taeniases and human cysticercosis. Moscow: Centre of International Projects GKNT; 1982. p. 208-14.

113. Keiser J, Utzinger J. Efficacy of current drugs against soil-transmitted helminth infections: systematic review and meta-analysis. JAMA. 2008;299(16):1937-48.

114. Kia EB, Masoud J, Yalda A, Mahmoudi M, Farahani H. Study on human taeniasis by administering anti-Taenia drug. Iranian Journal of Public Health. 2005;34(4):47-50.

115. Kihara T. Treatment of tapeworm infestation: a progress report. Saishin - Igaku. 1989;44(4):899-902.

116. Kimani BW, Mbugua AK, Kihara JH, Ng'ang'a M, Njomo DW. Safety, efficacy and acceptability of praziquantel in the treatment of Schistosoma haematobium in pre-school children of Kwale County, Kenya. PLoS neglected tropical diseases. 2018;12(10):e0006852.

117. Kociecka W. Intestinal cestodiases. Bailliere's Clinical Tropical Medicine and Communicable Diseases. 1987;2(3):677-94.

118. Komma MD, Santos VLV. Observations on Taenia solium (Linnaeus, 1758) and Taenia saginata (Goeze, 1782) from a group of 31 tapeworms, mostly removed with pumpkin seed taenifuge. Revista de Patologia Tropical. 1972;1(1):69-72.

119. Koul PA, Jeri C, Gilman RH, Lescano AG, Gonzalez AE, Garcia HH. Species identification after treatment for taeniasis [2] (multiple letters). Lancet (London, England). 2004;363(9425):1999.

120. Kurup R. Control of intestinal parasites among children in two communities of south Saint Lucia. Journal of Rural and Tropical Public Health. 2010;9:95-100.

121. Kurup R, Hunjan GS. Epidemiology and control of Schistosomiasis and other intestinal parasitic infections among school children in three rural villages of south Saint Lucia. Journal of vector borne diseases. 2010;47(4):228-34.

122. Kuzacute˜micki R, Gajda-Kazikowa E, Kolińska E. Preliminary comparative study of the efficacy of Radaverm and Yomesan in the treatment of taeniasis. Wiadomosci parazytologiczne. 1985;31(2):187-91.

123. Lara-Aguilera R, Aguilar-Bucio MT, Martinez-Toledo JL. [Taeniasis, amebiasis and other intestinal parasitosis in school age children from Michoacan State, Mexico]. Boletin medico del Hospital Infantil de Mexico. 1990;47(3):153-9.

124. Laranjo-Gonzalez M, Devleesschauwer B, Trevisan C, Allepuz A, Sotiraki S, Dorny P, et al. The epidemiology of Taenia Saginata and Taenia Solium in Western Europe: A systematic review. Tropical Medicine and International Health. 2017;22 (Supplement 1):189-90.

125. Latham MC, Stephenson LS, Hall A. Nutritional status, parasitic infections and health of road workers in 4 areas of Kenya: part IV. West Pokot District - the semi-arid highlands. East African medical journal. 1983;60(5):282-9.

126. Lazar L, Cristian-Davidescu A. [Management of taeniasis: comparative evaluation of osmotic non-oily purgatives]. Bacteriologia, virusologia, parazitologia, epidemiologia (Bucharest, Romania : 1990). 2004;49(3-4):151-6.

127. Levay J, Vilimszky Z. Recent progress in the therapy of human taeniasis. Magyar Allatorvosok Lapja. 1975;30(4):292-3.

128. Li H, Zhu J, Che Y. Efficacy of pumpkin seeds in combination with areca in treatment of 204 taeniasis cases of Blang ethnic group. China Tropical Medicine. 2013;13(8):1027-8.

129. Lightowlers MW, Garcia HH, Gauci CG, Donadeu M, Abela-Ridder B. Monitoring the outcomes of interventions against Taenia solium: options and suggestions. Parasite immunology. 2016;38(3):158-69.

130. Long C, Xiao N, Li T, Chen X, Zhou Z, Akira I, et al. Study of the effectiveness of drug combinations at treating taeniasis in the Tibetan regions of Sichuan, China. Zhongguo Bingyuan Shengwuxue Zazhi / Journal of Pathogen Biology. 2014;9(11):1000-3.

131. Louzada GZ. The treatment of taeniasis and hymenolepiasis with praziquantel. . 1979. p. [200].

132. Louzada GZ, Louzada FZ, Louzada TZ, Agra H, Louzada JLZ. Terapêutica da teníase e da himenolepíase com as doses mais eficazes de praziquantel segundo nossa experiência. Arq Bras Med. 1987;61(2):143-6.

133. Louzada GZ, Louzada FZ, Louzada TZ, Berlitz MF. Treatment of taeniasis and hymenolepiasis with praziquantel single oral dose. [Portuguese]. Folha Medica. 1979;79(4):323-6.

134. Louzada GZ, Louzada TZ, Louzada FZ, Berlitz MF. Praziquantel - A pilot study in the treatment of taeniasis and hymenolepiasis in humans. [Portuguese]. Folha Medica. 1978;77(1):41-4.

135. Madinga J, Kanobana K, Lukanu P, Abatih E, Baloji S, Linsuke S, et al. Geospatial and age-related patterns of Taenia solium taeniasis in the rural health zone of Kimpese, Democratic Republic of Congo. Acta tropica. 2017;165:100-9.

136. Magdieva SR. Aspects of the clinical course of taeniasis, its association with Enterobius and Giardia infections and phenasal treatment. Meditsinskaya Parazitologiya i Parazitarnye Bolezni. 1992(No. 5-6):23-5.

137. Margono SS, Wandra T, Swasono MF, Murni S, Craig PS, Ito A. Taeniasis/cysticercosis in Papua (Irian Jaya), Indonesia. Parasitology international. 2006;55 Suppl:S143-8.

138. Martin del Barco OH, Alvarez Manzanares P, Lopez Izquierdo R. Intestinal parasitosis. [Spanish]. FMC Formacion Medica Continuada en Atencion Primaria. 2009;16(1):14-24.

139. Martinez Baez M, Martinez Maranon R, Molina Pasquel C. Histological study of the proglottides of Taenia solium expelled after the administration of Yomesan. Revista de investigacion en salud publica. 1971;31(3):152-62.

140. Marty P, Mary C, Pagliardini G, Quilici M, Le Fichoux Y. [Brief survey on cysticercosis and Taenia solium taeniasis in a village of western Cameroon]. Medecine tropicale : revue du Corps de sante colonial. 1986;46(2):181-3.

141. McCleery EJ, Patchanee P, Pongsopawijit P, Chailangkarn S, Tiwananthagorn S, Jongchansittoe P, et al. Taeniasis among refugees living on Thailand-Myanmar border, 2012. Emerging infectious diseases. 2015;21(10):1824-6.

142. Mejia R, Bruschi F, Bottazzi ME. Global Health and Tropical Medicine in the Twenty-First Century: A Renewed Interest in the Understanding and the Control of Helminth Infections. Current Tropical Medicine Reports. 2015;2(4):238-40.

143. Misra PK, Pande NK, Jagota SC. Albendazole in the treatment of intestinal helminthiasis in children. Curr Med Res Opin. 1985;9(8):516-9.

144. Mohammed KA, Haji HJ, Gabrielli AF, Mubila L, Biswas G, Chitsulo L, et al. Triple co-administration of ivermectin, albendazole and praziquantel in zanzibar: a safety study. PLoS neglected tropical diseases. 2008;2(1):e171.

145. Montresor A, Palmer K. Taeniasis/cysticercosis trend worldwide and rationale for control. Parasitology international. 2006;55(SUPPL.):S301-S3.

146. Most H, Yoeli M, Hammond J, Scheinesson GP. Yomesan (niclosamide) therapy of hymenolepis nana infections. American Journal of Tropical Medicine and Hygiene. 1971;20(2):206-8.

147. Mwanjali G, Kihamia C, Kakoko DVC, Lekule F, Ngowi H, Johansen MV, et al. Prevalence and Risk Factors Associated with Human Taenia Solium Infections in Mbozi District, Mbeya Region, Tanzania. PLoS neglected tropical diseases. 2013;7 (3) (no pagination)(e2102).

148. Mwape KE, Phiri IK, Praet N, Muma JB, Zulu G, Van den Bossche P, et al. Taenia solium Infections in a rural area of Eastern Zambia-a community based study. PLoS neglected tropical diseases. 2012;6(3):e1594.

149. Nct. Taenia Solium Control Case Study in Zambia. Https://clinicaltrialsgov/show/nct01368354. 2011.

150. Nct, Gabriel S, Mwape E. Taenia Solium Elimination Versus Control: what is the Best Way Forward for Sub-Saharan Africa? Https://clinicaltrialsgov/show/nct02612896. 2015.

151. Neghina R, Neghina AM, Marincu I, Iacobiciu I. Human taeniasis in western Romania and its relationship to multicultural food habits and influences. Foodborne pathogens and disease. 2010;7(5):489-92.

152. Njomo DW, Tomono N, Muhoho N, Mitsui Y, Josyline KC, Mwandawiro CS. The adverse effects of albendazole and praziquantel in mass drug administration by trained schoolteachers. African Journal of Health Sciences. 2010;17(3-4):10-4.

153. Nwosu ABC. Intestinal helminthiases in Nigeria: Epidemiology, infection dynamics, and control strategies. [French]. Medecine d'Afrique Noire. 1982;29(Spec.):49-53.

154. Nwosu ABC. Intestinal helminthiases in Nigeria: epidemiology, infection dynamics, and control strategies. Medecine d'Afrique Noire. 1982;29(8/9):587-91.

155. O'Neal SE, Gavidia C, Gamboa R, Muro C, Vilchez P, Moyano LM, et al. Ring strategy as an effective alternative to mass drug administration for control of taenia solium taeniasis/cysticercosis. American Journal of Tropical Medicine and Hygiene. 2017;97 (5 Supplement 1):12.

156. Ofori-Adjei D, Dodoo AN, Appiah-Danquah A, Couper MR. A review of the safety of niclosamide, pyrantel, triclabendazole and oxamniquine. International Journal of Risk and Safety in Medicine. 2008;20(3):113-22.

157. Ohnishi K, Sakamoto N, Kobayashi K, Iwabuchi S, Nakamura-Uchiyama F. Therapeutic effect of praziquantel against Taeniasis asiatica. International journal of infectious diseases : IJID : official publication of the International Society for Infectious Diseases. 2013;17(8):e656-7.

158. Okello AL, Thomas LF. Human taeniasis: current insights into prevention and management strategies in endemic countries. Risk management and healthcare policy. 2017;10:107-16.

159. Okelo GBA. Open and placebo-controlled studies of albendazole in the treatment of intestinal helminthiasis. London: Royal Society of Medicine; 1984. p. 57-62.

160. Olveda RM, Icatlo Jr FC, Libranda d LB. A community-based clinical trial of Albendazole in Leyte, Philippines. Philippine Journal of Internal Medicine. 1983;21(3):126-33.

161. Onkanga IO, Mwinzi PN, Muchiri G, Andiego K, Omedo M, Karanja DM, et al. Impact of two rounds of praziquantel mass drug administration on Schistosoma mansoni infection prevalence and intensity: a comparison between community wide treatment and school based treatment in western Kenya. International journal for parasitology. 2016;46(7):439-45.

162. Patwari A, Aneja S, Singh G, Manhas RS. A study of taeniasis in children. Indian pediatrics. 1980;17(6):515-7.

163. Pawlowski Z, Allan J, Sarti E. Control of Taenia solium taeniasis/cysticercosis: from research towards implementation. International journal for parasitology. 2005;35(11-12):1221-32.

164. Pawlowski ZS. Large-scale use of chemotherapy of taeniasis as a control measure for Taenia solium infections. 1987. In: Helminth Zoonoses [Internet]. Dordrecht: Martinus Nijhoff PublishersCurrent Topics in Veterinary Medicine and Animal Science; [100-5]. Available from: https://www.springer.com/gp/book/9780898388961.

165. Pawlowski ZS. Perspectives on the control of Taenia solium. Parasitology Today. 1990;6(12):371-3.

166. Pawlowski ZS. Control of Taenia solium taeniasis and cysticercosis by focus-oriented chemotherapy of taeniasis. The Southeast Asian journal of tropical medicine and public health. 1991;22 Suppl:284-6.

167. Pawlowski ZS. Role of chemotherapy of taeniasis in prevention of neurocysticercosis. Parasitology international. 2006;55 Suppl:S105-9.

168. Pawłowski ZS. Efficacy of low doses of praziquantel in taeniasis. Acta tropica. 1990;48(2):83-8.

169. Pawłowski ZS. Efficacy of low doses of praziquantel in taeniasis. Acta tropica. 1991;48(2):83-8.

170. Pearson RD, Guerrant RL. Praziquantel: a major advance in anthelminthic therapy. Annals of internal medicine. 1983;99(2):195-8.

171. Pearson RD, Hewlett EL. Niclosamide therapy for tapeworm infections. Annals of internal medicine. 1985;102(4):550-1.

172. Pene P, Mojon M, Garin JP. Albendazole: A new broad spectrum anthelmintic. Double-blind multicenter clinical trial. American Journal of Tropical Medicine and Hygiene. 1982;31(2):263-6.

173. Penrith ML. Cysticercosis Working Group in Eastern and Southern Africa--6th General Assembly. Journal of the South African Veterinary Association. 2009;80(4):206-7.

174. Perera DR, Western KA, Schultz MG. Niclosamide treatment of cestodiasis. Clinicial trials in the United States. The American journal of tropical medicine and hygiene. 1970;19(4):610-2.

175. Pierattelli M. Intestinal parasitosis. [Italian]. Occhio Clinico Pediatria. 2001;5(7):19-21.

176. Plotnikov NN, Karnaukhov VK, Ozeretskovskaya NN, Stromskaya TF, Firsova RA. Clinical trials of cucurbitine (preparation of pumpkin seeds) in cestodiases. Meditsinskaya Parazitologiya i Parazitarnye Bolezni. 1972;41(4):407-11.

177. Prasad KN, Prasad A, Gupta RK, Pandey CM, Singh U. Prevalence and associated risk factors of Taenia solium taeniasis in a rural pig farming community of north India. Transactions of the Royal Society of Tropical Medicine and Hygiene. 2007;101(12):1241-7.

178. Radoev V. Treatment of teniasis (Bulgarian). [Bulgarian]. EpidemMikrobiolInfekBolesti. 1974;11(1):72-4.

179. Radomyos P, Bunnag D, Harinasuta T. Worms recovered in stools following praziquantel treatment. Arzneimittel-Forschung/Drug Research. 1984;34(9 B):1215-7.

180. Rajshekhar V. Purging the worm: management of Taenia solium taeniasis. Lancet (London, England). 2004;363(9413):912.

181. Ramos-Zúñiga R, Pérez-Gómez HR, Jáuregui-Huerta F, López-Hernández MdS, Valera-Lizárraga JE, Paz-Vélez G, et al. Incidental consequences of antihelmintic treatment in the central nervous system. World neurosurgery. 2013;79(1):149-53.

182. Raso G, N'Goran EK, Toty A, Luginbuhl A, Adjoua CA, Tian-Bi NT, et al. Efficacy and side effects of praziquantel against Schistosoma mansoni in a community of western Cote d'Ivoire. Transactions of the Royal Society of Tropical Medicine and Hygiene. 2004;98(1):18-27.

183. Richards F, Jr., Schantz PM. Treatment of Taenia solium infections. Lancet (London, England). 1985;1(8440):1264-5.

184. Rim HJ, Lee JS, Joo KH. Therapeutic effects of praziquantel (EMBAY 8440) against taeniasis and hymenolepiasis nana. Korean Journal of Parasitology. 1978;16(1):60-1.

185. Rodriguez-Canul R, Fraser A, Allan JC, Dominguez-Alpizar JL, Argaez-Rodriguez F, Craig PS. Epidemiological study of Taenia solium taeniasis/cysticercosis in a rural village in Yucatan state, Mexico. Annals of tropical medicine and parasitology. 1999;93(1):57-67.

186. Rossignol JF. Albendazole: Clinical study in France and West Africa. A 1034 cases report. [Spanish]. Compendium de Investigaciones Clinicas Latinoamericanas. 1981;1(Suppl. 1):117-25.

187. Rossignol JF, Maisonneuve H. Albendazole: placebo-controlled study in 870 patients with intestinal helminthiasis. Transactions of the Royal Society of Tropical Medicine and Hygiene. 1983;77(5):707-11.

188. Rossignol JF, Maisonneuve H. Albendazole: A new concept in the control of intestinal helminthiasis. Gastroenterologie Clinique et Biologique. 1984;8(6-7):569-76.

189. Sanchez AL, Gomez O, Allebeck P, Cosenza H, Ljungstrom L. Epidemiological study of Taenia solium infections in a rural village in Honduras. Annals of tropical medicine and parasitology. 1997;91(2):163-71.

190. Sanchez AL, Medina MT, Ljungstrom I. Prevalence of taeniasis and cysticercosis in a population of urban residence in Honduras. Acta tropica. 1998;69(2):141-9.

191. Santana IMd, Silva GRd, Marques SR, Costa ACMdSFd, Alves LC, Faustino MAdG. Analysis of the occurrence of human geohelmintiasis in communities of the Metropolitan Region of Recife-PE - preliminary results. Ciência Veterinária nos Trópicos. 2015;18(2):151-3.

192. Sarti E, Rajshekhar V. Measures for the prevention and control of Taenia solium taeniosis and cysticercosis. Acta tropica. 2003;87(1):137-43.

193. Sarti E, Schantz P, Flisser A. Evaluation of two intervention strategies for the prevention and control of Taenia solium cysticercosis in rural areas of Mexico. Parasitology international. 1988;47(Suppl.):77.

194. Sarti E, Schantz PM, Plancarte A, Wilson M, Gutierrez OI, Aguilera J, et al. Epidemiological investigation of Taenia solium taeniasis and cysticercosis in a rural village of Michoacan state, Mexico. Transactions of the Royal Society of Tropical Medicine and Hygiene. 1994;88(1):49-52.

195. Sato MO, Sato M, Yanagida T, Waikagul J, Pongvongsa T, Sako Y, et al. Taenia solium, Taenia saginata, Taenia asiatica, their hybrids and other helminthic infections occurring in a neglected tropical diseases' highly endemic area in Lao PDR. PLoS neglected tropical diseases. 2018;12 (2) (no pagination)(e0006260).

196. Shafei AZ. Effects of niclosamide. [Portuguese]. Hospital (Rio de Janeiro). 1965;67(6):1307-11.

197. Singh HL, Singh NB, Singh YI. Helminthic infestation of the primary school-going children in Manipur. Journal of Communicable Diseases. 2004;36(2):111-6.

198. Sircar AD, Mwinzi PNM, Onkanga IO, Wiegand RE, Montgomery SP, Secor WE. Schistosoma mansoni Mass Drug Administration Regimens and Their Effect on Morbidity among Schoolchildren over a 5-Year Period-Kenya, 2010-2015. The American journal of tropical medicine and hygiene. 2018;99(2):362-9.

199. Sobota K. [Our experiences with the treatment of teniasis]. Bratislavske lekarske listy. 1969;51(2):213-9.

200. Soh CT, Lee JH, Kim SH. Clinical trial with niclosamide in Taenia infection. Korean Journal of Parasitology. 1976;14(2):180.

201. Soh CT, Min DY, Lee JH, Kim SH. Treatment of taeniasis with Niclosamide. Yonsei Reports on Tropical Medicine. 1976;7(1):74-6.

202. Stahel E. Treatment of intestinal parasites. [German]. Deutsche Medizinische Wochenschrift. 1977;102(4):133-5.

203. Steinmann P, Yap P, Utzinger J, Du ZW, Jiang JY, Chen R, et al. Control of soil-transmitted helminthiasis in Yunnan province, People's Republic of China: Experiences and lessons from a 5-year multi-intervention trial. Acta Tropica. 2014;30.

204. Suroso T, Margono SS, Wandra T, Ito A. Challenges for control of taeniasis/cysticercosis in Indonesia. Parasitology international. 2006;55(SUPPL.):S161-S5.

205. Tanowitz HB, Weiss LM, Wittner M. Diagnosis and treatment of intestinal helminths. I. Common intestinal cestodes. The Gastroenterologist. 1993;1(4):265-73.

206. Tanowitz HB, Weiss LM, Wittner M. Tapeworms. Current infectious disease reports. 2001;3(1):77-84.

207. Thi Thi H, Thanda T, Khin Yi O, Win T, Htay Htay T, Chai J, et al. Status of infection with soil-transmitted helminths among primary school children in three selected townships of Yangon Region. Myanmar Health Sciences Research Journal. 2015;27(3):221-6.

208. Tianzuan C, Hongxie X, Binghong W. Clinical therapeutic analysis on 147 cases of taeniasis. Chinese journal of infectious diseases. 1991;9(Suppl 4):236‐.

209. Todorov R. Our experience in the treatment of taeniasis. Savremenna Medicina. 1973;24(10):22-4.

210. Torres M, Perez C, Galdamez E, Gabor M, Miranda C, Cofre X, et al. Teniosis: Serie clínica en 35 pacientes. Parasitología al día. 2001;25(1-2):55-9.

211. Van De N, Le TH, Lien PT, Eom KS. Current status of taeniasis and cysticercosis in Vietnam. The Korean journal of parasitology. 2014;52(2):125-9.

212. Vidal LMM, Gamboa R, Vilchez P, Ayvar V, Atto R, Oliva E, et al. Efficacy of single doses of praziquantel 5-10 MG/KG for taeniasis under controlled conditions in rural communities of the northern coast of Peru. American Journal of Tropical Medicine and Hygiene. 2016;95 (5 Supplement 1):141.

213. Vitta A, Polseela R, Bunchu N, Waree P, Thongwat D, Tangchaisuriya U, et al. Intestinal helminthiases in two communities of Phitsanulok province, northern Thailand. Journal of Tropical Medicine & Parasitology. 2012;35(1):1-5.

214. Wandra T, Depary AA, Sutisna P, Margono SS, Suroso T, Okamoto M, et al. Taeniasis and cysticercosis in Bali and North Sumatra, Indonesia. Parasitology international. 2006;55(SUPPL.):S155-S60.

215. Wandra T, Sudewi AA, Swastika IK, Sutisna P, Dharmawan NS, Yulfi H, et al. Taeniasis/cysticercosis in Bali, Indonesia. The Southeast Asian journal of tropical medicine and public health. 2011;42(4):793-802.

216. Watts NS, Pajuelo M, Clark T, Loader MC, Verastegui MR, Sterling C, et al. Taenia solium infection in Peru: a collaboration between Peace Corps Volunteers and researchers in a community based study. PloS one. 2014;9(12):e113239.

217. Wilson JM, Howarth SE, Ravaoalimalala V, Ranaivoson E, Crook S, Denning A, et al. A study of bilharzia and intestinal worms in Morondava. Archives de l'Institut Pasteur de Madagascar. 1987;53(1):105-16.

218. Xiao SH, Utzinger J, Tanner M, Keiser J, Xue J. Advances with the Chinese anthelminthic drug tribendimidine in clinical trials and laboratory investigations. Acta tropica. 2013;126(2):115-26.

219. Yap P, Wu FW, Du ZW, Hattendorf J, Chen R, Jiang JY, et al. Effect of Deworming on Physical Fitness of School-Aged Children in Yunnan, China: A Double-Blind, Randomized, Placebo-Controlled Trial. PLoS neglected tropical diseases. 2014;8 (7) (no pagination)(e2983).

220. Zhang SF. [Treatment of 60 cases with Taenia solium infection by praziquantel]. Zhongguo ji sheng chong xue yu ji sheng chong bing za zhi = Chinese journal of parasitology & parasitic diseases. 2003;21(4):196.

221. Zhang WZ. Treatment of 150 cases of taeniasis. Journal of Parasitology and Parasitic Diseases. 1985;3(4):271.
