# Supplementary material for: Systematic review of the effectiveness of selected drugs for preventive chemotherapy for Taenia solium taeniasis
Source: PLoS Negl Trop Dis. 2020 Jan 16;14(1):e0007873. doi: 10.1371/journal.pntd.0007873 (PMC6964831; doi:10.1371/journal.pntd.0007873)
Supplement: S4 File — (DOCX) [file pntd.0007873.s005.docx]

# S4 File. Side-effects reported in studies found through supplementary searching of treatment with albendazole, niclosamide or praziquantel of other parasites

## Supplementary search for studies of side-effects with albendazole, niclosamide or praziquantel

Given the limited evidence on side-effects from studies that met the inclusion criteria for the review, we conducted a supplementary search for studies reporting side-effects from treatment with NICL, PZQ or ALB of taeniasis (but not yet published) or of other parasites, especially in *Taenia solium* endemic (or suspected-endemic) countries. These studies were found from the list of excluded studies from the systematic review as well as searching of Google with the keywords: (niclosamide, praziquantel and/or albendazole) and (side-effect or adverse). It is important to note that this search was not systematic, and no quality assessment of the studies was conducted.

We found 12 additional studies, which are summarized in the supplementary table on side-effects below (1-12). With the exception of the three studies that reviewed adverse effects databases (2, 4, 10), all of the studies were conducted in *Taenia solium* endemic countries or, where there were multiple countries, most were *Taenia solium* endemic countries. In summary, the findings for each drug are:

ALB – Most studies included in the table reported only mild and transient side-effects, a hospital-based study of admissions to hospital in India following National De-Worming Day identified 25 children and adolescents (<0.03% of the population) with 64 adverse events, of which 20 events were severe and 11 were very serious, including loss of consciousness in 8 and convulsions in 3 (1). A multi-country randomized, placebo-controlled trial of ALB for soil-transmitted helminths at 400mg for adults and 200mg for children (n=870, all ages 3-79 years) showed no significant difference between the two groups (12).

NICL – One large study of MDA for taeniasis in Peru (all ages ≥ 2 years), using a dose of 2g (adjusted for children) involved 158,201 doses administered over 3 rounds of MDA with home visits after 72 hours to assess adverse events (3). There were 1418 reports (0.9%) of adverse events, all of mild intensity. None were severe. The review of the adverse events database in France (non-endemic for *Taenia solium*) found 7 reports associated with tapeworm infection – mostly digestive disorders, one skin reaction and one anaphylactoid reaction leading to hospitalization. The review of the Uppsala Monitoring Centre Database (most countries included were non-endemic) had 82 reports (involving 173 reactions) between 1971 and May 2006 with NICL (doses not specified) (10). These involved skin and appendages (43), gastrointestinal tract (38) and cardiovascular system (28) and there were 9 reports of anaphylactic shock and anaphylactoid reactions.

PZQ – doses of 40mg/kg were used for MDA for Schistosomiasis in Uganda (all ages) (5), Kenya (pre-school age and school-aged children) (7), and Côte d’Ivoire (all ages) (11). All of these countries are *Taenia solium* endemic. Almost all studies showed that the side-effects were mild and transient, occurring in the first hours after treatment and gradually resolving within 24 hours. No cases of neurocysticercosis are reported in these studies nor in the reviews of adverse events databases conducted in France and Korea, though there was one case of an allergic reaction (France) (2) and 2 serious adverse events that weren’t specified (Korea) (4).

## References

1. Agrawal P, Srivastava B, Bhardwaj R, Gaur S. Adverse events of albendazole due to mass drug administration. International Journal of Basic & Clinical Pharmacology. 2017;6(7):1674-7.

2. Bagheri H, Simiand E, Montastruc J-L, Magnaval J-F. Adverse Drug Reactions to Anthelmintics. Annals of Pharmacotherapy. 2004;38(3):383-8.

3. Gamboa R, Vilchez P, Moyano LM, Muro C, Benavides V, O’Neil SE, et al. Efficacy and adverse events of niclosamide in a large scale cysticercosis elimination demonstration program on the North Coast of Peru. American Journal of Tropical Medicine and Hygiene. 2017;95(5 Suppl):140.

4. Hong ST. Albendazole and Praziquantel: Review and Safety Monitoring in Korea. Infect Chemother. 2018;50(1):1-10.

5. Kabatereine NB, Kemijumbi J, Ouma JH, Sturrock RF, Butterworth AE, Madsen H, et al. Efficacy and side effects of praziquantel treatment in a highly endemic Schistosoma mansoni focus at Lake Albert, Uganda. Transactions of the Royal Society of Tropical Medicine and Hygiene. 2003;97(5):599-603.

6. Keiser J, Utzinger J. Efficacy of current drugs against soil-transmitted helminth infections: systematic review and meta-analysis. JAMA. 2008;299(16):1937-48.

7. Kimani BW, Mbugua AK, Kihara JH, Ng'ang'a M, Njomo DW. Safety, efficacy and acceptability of praziquantel in the treatment of Schistosoma haematobium in pre-school children of Kwale County, Kenya. PLoS neglected tropical diseases. 2018;12(10):e0006852.

8. Mohammed KA, Haji HJ, Gabrielli AF, Mubila L, Biswas G, Chitsulo L, et al. Triple co-administration of ivermectin, albendazole and praziquantel in zanzibar: a safety study. PLoS neglected tropical diseases. 2008;2(1):e171.

9. Njomo DW, Tomono N, Muhoho N, Mitsui Y, Josyline KC, Mwandawiro CS. The adverse effects of albendazole and praziquantel in mass drug administration by trained schoolteachers. African Journal of Health Sciences. 2010;17(3-4):10-4.

10. Ofori-Adjei D, Dodoo AN, Appiah-Danquah A, Couper MR. A review of the safety of niclosamide, pyrantel, triclabendazole and oxamniquine. International Journal of Risk and Safety in Medicine. 2008;20(3):113-22.

11. Raso G, N'Goran EK, Toty A, Luginbuhl A, Adjoua CA, Tian-Bi NT, et al. Efficacy and side effects of praziquantel against Schistosoma mansoni in a community of western Cote d'Ivoire. Transactions of the Royal Society of Tropical Medicine and Hygiene. 2004;98(1):18-27.

12. Rossignol JF, Maisonneuve H. Albendazole: placebo-controlled study in 870 patients with intestinal helminthiasis. Transactions of the Royal Society of Tropical Medicine and Hygiene. 1983;77(5):707-11.

## Table. Side-effects reported in studies found through supplementary searching of treatment with albendazole, niclosamide or praziquantel of other parasites

| **Author, year, Country & Study type** | **Age group** | **Drug and dose**  **Treatment for what disease/parasite?** | **How was information on side-effects measured / collected?** | **Number treated** | **Number with side-effects** | **Describe side-effects** |
| --- | --- | --- | --- | --- | --- | --- |
| Agrawal et al. 2017 (1)  India  Cross-sectional, hospital-based | Children & adolescents: 2-19 years | ALB - 1 tablet  MDA for soil-transmitted helminths | Questionnaire to parents/children admitted to hospital (Govt Medical College Haldwani) for adverse events due to ALB administration for national de-worming programme. Categorized as mild, moderate, or severe based on WHO guidelines 2011 | All children treated on National De-Worming Day and following 4 days. Haldwani has a population of over 200,000. | 25 (<0.03% of population) | 25 children experienced 64 adverse events, of which 21 events were mild, 12 were moderate, 20 were severe and 11 were serious. Average duration of stay in hospital was 3.4 days. Mean onset of symptoms was 5.6 hours with standard deviation of 1.5 hours. Abdominal pain was reported by 19 (76%), headache by 11 (44%), loss of consciousness by 8 (32%), vomiting by 7 (28%), nausea by 4 (16%), convulsions by 3 (12%), rashes by 2 (8%), fever by 2 (8%), breathlessness by 1 (4%) and vertigo by 1 (4%). |
| Bagheri et al. 2004 (2)  France  Retrospective study - Review of database | All ages | ALB, NICL and PZQ are reported here  **ALB:** prescribed for treatment of larval taeniid infections (n=8, dosages of 800 to 3200 mg/day), echinococcosis (n = 5), cysticercosis (n = 2), and strongyloïdiasis (n = 1). In 20 ALB was used in lesser dosages (400–800 mg/day) for toxocariasis (n = 17), mycobacterial infection (n = 1), and microsporidiosis (n = 1). 2 not specified. **NICL:** 7 cases of tapeworm infections, 1 for echinococcosis and 1 for unknown reason. **PZQ:** 12 for schistosomiasis treatment. | The information is based on that reported to the French Pharmacovigilance Database (FPD) to identify all anthelmintic-induced adverse effects collected by spontaneous reporting. The information presented is from January 1985-August 1999 (14 years). | N/A | 243 patients with 291 suspected AE. Of these, 29 cases (34 AE were due to ALB use; 9 cases (9 AE) due to NICL use; and 16 cases (16 AE) due to PZQ use. | **ALB:** Hematologic AE occurred in 4 cases, hepatitis in 3, and acute renal failure in another case. For 6 patients, AE were classified as serious leading to hospitalization: 2 cases of agranulocytosis, 1 of hepatitis, 1 of retrobulbar neuritis, 1 of acute renal failure, and 1 of skin rash. **NICL:** AE occurring during taeniasis treatment were digestive disorders (abdominal pain, vomiting) for 3 cases, dizziness for 2 cases, skin reactions (polymorph erythema) and sweating for one case, respectively, and one anaphylactoid reaction leading to hospitalization after a single intake of the drug. Niclosamide use for echinococcosis elicited a benign erythema. **PZQ:** PZQ was prescribed in 12 cases for schistosomiasis. In 1 case, an allergic reaction led to hospitalization and was classified as serious. |
| Gamboa et al. 2017^a^ (3)  Peru  Before-after study | All ages ≥ 2 years | NICL - children with 11 to 34 kg: 1g, 34 to 49 kg: 1.5g, ≥ 50kg: 2g  MDA for taeniasis | Home visits 72 hours after treatment to assess adverse events | At total of 158,201 doses were administered across all 3 rounds (between June 2009 – April 2010) with 68,751 (86.8%) people receiving at least one dose. | 1418 (0.9%) reported events related to treatment. | The prevalence of AE related to ingestion of NICL was 0.90% (1418/158,201), of which 98.73% (1400/1418) were of mild intensity. There were no severe AE. Abdominal pain was the most frequently reported adverse event (571/1418; 40.27%). AE were more common among females [PR=2.33; 95% CI 2.06-2.65] and among those that received NICL in more than one treatment round [2 doses PR 1.18; 95% CI 0.97-1.44; 3 doses PR 1.46; 95% CI 1.27-1.67). |
| Hong 2018 (4)  Korea  Retrospective study - Review of database | All ages | ALB and PZQ at various doses.  **ALB:** for STH or intestinal helminths (Ascaris, hookworm, Trichuris, Strongyloides, Enterobius), and for tissue helminths (Trichinella, Toxocara, Echinococcus, cysticercus, Filaria, and Onchocerca). **PZQ:** for intestinal trematodes and cestodes, and for tissue helminths (Clonorchis/Opisthorchis Paragonimus, Echinococcus, and neurocysticercosis | It was collected (downloaded) from The Korea Institute of Drug Safety & Risk Management (KIDS), which monitors drug safety related events and collects nation-wide reports. | N/A. However, the author reports an estimate of 152,000 Korean people treated with PZQ during 2012-15. Given that the study spans 9 years and that it includes ALB, the number treated is likely to be substantially more. | For 2006-2015: a total of 856 reports were listed with ALB or PZQ; many overlapped with multiple complaints. Specifically, 256 cases were associated with ALB and 108 with PZQ | Of the total of cases reported (256) as showing AE due to **ALB** administration, only 1 case could be verified as genuine, 45 as probable, 76 as possible and 134 as unlikely or not assessable; for **PZQ** of 108 cases reported only 1 case of AE was attributed to the drug, 27 as probable, 35 as possible and 45 as unlikely or not assessable. Most of the reported AE were mild and transient, and subsided spontaneously and rapidly. The most frequent symptoms induced by both ALB and PZQ were vomiting and nausea, but several other AE that are often seen following anthelmintic treatment were also observed. There were 6 cases regarded as showing serious AE, 6 following ALB and the other 2 after PZQ treatment. In 4 of these cases (3 ALB, 1 PZQ), in addition to the administration of the anti-parasites, the patients had been receiving concomitantly several other drugs. |
| Kabatereine et al. 2003 (5)  Uganda  Before-after study | All ages: 5-54 years | PZQ - 40 mg/kg; repeated 6 weeks later  Selective chemotherapy for *Schistosoma mansoni* | The symptom profile pre-and post-treatment was based on interviews (346 individuals). | 617 randomly selected to represent all ages and both genders, of which 482 were infected and treated. | 272 (79%, 272/346) | Abdominal pain, diarrhea, dizziness, vomiting, fatigue, body rash, and nausea occurred with a significantly higher frequency after treatment than before treatment, while fever and headache, which were common before treatment, significantly declined. Most of the side effects developed between 30 min and 6 h after treatment but had subsided within 24 h. None of the side effects were so severe that they warranted steroid treatment. |
| Keiser et al. 2008 (6)  Various countries  Systematic review of RCTs | All ages: children or adults | ALB 400mg  Selective chemotherapy for STH | "Some trials failed to report whether adverse events were monitored at all, and safety measures overall lacked quality." | 14 RCTS of ALB including 557 individuals, of which 12 studies contributed data on side-effects. | 3 individuals | Albendazole was well tolerated. In 11 studies included in our meta-analysis, no significant AE were reported following albendazole administration. One trial carried out in the Philippines reported nausea and diarrhea in 2 and 1 individuals, respectively. There was no indication whether or not AE were assessed in the remaining 2 RCTs. |
| Kimani et al. 2018 (7)  Kenya  Before-after study | Pre-school children ≤ 6 years of age (mostly 3-6 years, only one child aged 2 years) | PZQ - 40 mg/kg  Selective chemotherapy for Schistosoma haematobium | 1h post-treatment AE were recorded by the teachers and community health extension workers who took part in the deworming exercise. Parents or guardians also interviewed using questionnaires 24h post-treatment for episodes of treatment-related AE. The study clinician evaluated the following AE abdominal pain, dizziness, nausea, headache, vomiting, drowsiness, itching, as likely or unlikely associated with study drug. Other symptoms reported by parents or guardians were also recorded. | 400, of which 330 were assessed for adverse effects | 10 (3%, 10/320) | 330/400 of children (82.5%) recruited in the study were assessed for adverse effects: 1 experienced dizziness, 1 had a headache, 4 had abdominal pain/discomfort, 2 had nausea and 2 experienced itching. None of the children vomited. There were no severe adverse events. While six respondents took no action when their child experienced an adverse event, one gave food, two gave milk and the other one made the child rest. |
| Mohammed et al. 2008 (8)  Tanzania  Cross-sectional  Note: The area where the study was conducted has very few pigs, thus unlikely to have *Taenia solium*. | All ages > 5 years | 3 treatments given at the same time: 1) ivermectin (200 µg/kg); 2) ALB (400 mg); and 3) PZQ (40 mg/kg)  MDA for lymphatic filariasis (ivermectin), STH (ALB) and Schistosomiasis (PZQ) | Participants were invited to report to participant first-line centres in the event of any side-effects (‘‘anything abnormal occurring in your body’’), health centre personnel were trained and instructed to refer to second-line hospitals any individual presenting with side effects that they could not manage. All treated individuals (for pilot study and a subsample for the full study) were interviewed between 5 and 7 days after treatment on occurrence of side-effects by experienced professional health staff and/or researchers. | 5055 were treated in an initial pilot study; a follow up study included 700,000 people, of which 19,043 were interviewed. | In the pilot study 504/5055 (10%) reported side effects; in the follow-up study 266/19,043 (1.4%) reported side effects. | **Pilot study:** Only 1 treated individual reported to a first-line health centre complaining about vomiting following treatment; symptoms were, however, mild ... and did not require referral to a second-line hospital facility. Interviews carried between day 5 and 7 post treatment showed a total of 615 events were reported by 504 individuals. 87.3% of symptoms occurred within 24h of treatment, 11.9% on the second day, and 0.8% on the third day. The symptom most frequently reported was dizziness. All symptoms were reported to be mild and subsided within a period of 24 hours after onset. **Follow-up study:** Overall, only 266 individuals (1.4%) of the interviewees reported any side-effects, none of which was judged to be significant enough to justify a visit to the nearest health centre. All the side-effects were mild, the most frequent being fatigue (n = 102), abdominal pain (n = 67), dizziness (n = 57), fever (n = 27) and vomiting (n = 13). These side-effects were accepted and managed by those who reported them. They were transient and all continued for less than 24h. |
| Njomo et al. 2010 (9)  Kenya  Controlled trial | School aged children | Two treatment groups: Mwea (intervention) - ALB 400 mg and PZQ 40 mg/kg; Ndia (control) - ALB 400 mg  MDA for soil-transmitted helminths (ALB) and *Schistosoma mansoni* (PZQ) | 2 schools from Ndia (ALB) and 3 from Mwea (ALB + PZQ) targeted for monitoring of adverse effects. Questionnaire distributed to randomly selected pupils one week after MDA – completed by pupils with guidance of the teachers. | Not reported. | 108/259: 49.7% in Mwea vs 39.2% % by pupils of Ndia. | For the ALB group, the most frequent adverse effect was stomach ache (13.4%), followed by headache (5.5%). Coughing, which is not a side effect of taking the drugs and could have been due to any other cause, was reported by (16.1%) of the pupils from Ndia. For the ALB + PZQ group, the most frequent adverse effect was stomach ache (30.1%), followed by vomiting/nausea (8.2%), headache (5.5%), and dizziness (5.5%). All adverse effects were temporary, and none were severe. |
| Ofori-Adjey et al. 2008 (10)  Various countries  Retrospective study - Review of literature and UMC database | Not reported | NICL.  NICL is retained in the WHO Model List of Essential Medicines 2005 and WHO Model Formulary 2006 for treating *T. saginata*, *T. solium*, *Diphyllobothrium latum*, *Hymenolepis nana* and *Dipylidium caninum* infections. | Adverse drug reaction reports in the Uppsala Monitoring Centre (UMC) database of the WHO Collaborating Centre for International Drug Monitoring in Sweden. Plus literature review. | N/A | 82 reports | The AE of NICL include the following: nausea, retching, abdominal pain, lightheadedness and pruritus (WHO Model Formulary 2006). Since 1971, there have been 82 reports of suspected adverse drug reactions to NICL in the WHO UMC database. These 82 reports from 16 countries involve 173 reactions from 16 countries. The most common adverse reactions are those involving the skin and appendages (43), gastrointestinal tract (38) and cardiovascular system (28). There were 9 reports of anaphylactic shock and anaphylactoid reactions. (UMC database accessed on 23/05/06). |
| Raso et al. 2004 (11)  Côte d'Ivoire  Before-after study | All ages | PZQ 40 mg/Kg for *Schistosoma mansoni*  Selective chemotherapy for *Schistosoma mansoni*. There was a follow-up survey 6 wk later. Individuals who continued to excrete *S. mansoni* eggs in their faeces at this time were treated with another dose of PZQ at 40 mg/Kg | Individuals treated with PZQ were instructed to report side effects within 24h post-treatment. The list of symptoms included nausea, vomiting, abdominal pain, diarrhoea, itching, headache, urticaria and dizziness. | 200 | 25 (12.5%, 25/200) | Among the 200 treated individuals, 25 reported 1 or more side effects within 24 h post-treatment. Side effects were mild and transient, i.e. they occurred in the first hours after treatment and gradually resolved within 24 hours. A higher proportion of individuals with high infection intensities reported diarrhoea (χ2 = 16.85, d.f. = 2, P < 0.001) and dizziness (Fisher’s exact test, P =0.036) when compared to individuals with light or moderate infections. None of the other side effects showed an association with infection intensity, and side effects showed no association with age categories. |
| Rossignol et al. 1983 (12)  Multi-regional study involving 11 countries  Randomized, placebo-controlled trial | All ages: 3-79 years | ALB 200 and 100 mg twice daily for adults and children under 12 years, respectively. When mouth inspection was impossible in the evening (in 49% of the total number of cases), the daily dose was given once as a 400 and 200 mg for adults and children, respectively.  Selective chemotherapy for STH | Each patient was interviewed and underwent a complete physical examination on the initial visit ... The same physical examination ... was carried out 24-72 h after the last treatment, and each patient was carefully questioned about side effects. | Of the 870 patients treated, 457 received ALB and 413 received a placebo | The total number of cases that reported side-effects was 55 (12%) for the ALB group vs 44 (11%) for the placebo. | Side-effects reported by patients treated with ALB vs. placebo are as follow: dizziness (3 vs 5); headache (8 vs 10); epigastric pain (30 vs 22); dry mouth (1 vs 0); fever (1 vs 1); pruritus (2 vs 1); vomiting (2 vs 1); and diarrhoea (8 vs 4). The total number of cases was 55 for ALB vs 44 for placebo. There was no significant difference between the number of signs reported in the two groups (χ2 = 0.415, N.S.). |

AE - adverse effects/events; ALB - albendazole; ECDE - Early Childhood Development Education; MDA – mass drug administration; NA - not applicable; NICL - niclosamide; PZQ - praziquantel; RCT - randomized controlled trial; SC – selective chemotherapy; STH - soil-transmitted helminths

a – this study is for treatment of taeniasis but is included here because it is a preliminary report of a study that is likely to meet the full inclusion criteria for the systematic review when published in full.
