# Supplementary material for: Systematic review of the effectiveness of selected drugs for preventive chemotherapy for Taenia solium taeniasis
Source: PLoS Negl Trop Dis. 2020 Jan 16;14(1):e0007873. doi: 10.1371/journal.pntd.0007873 (PMC6964831; doi:10.1371/journal.pntd.0007873)

## S1 Fig. Risk of bias graphs: review authors' judgements about each risk of bias item for each included study. Panel A, studies with a control group. Panel B, before-after studies.

Green circles represent low risk of bias, yellow circles unclear risk of bias, and red circles high risk of bias.

**A. Studies with a control group B. Before-after studies**


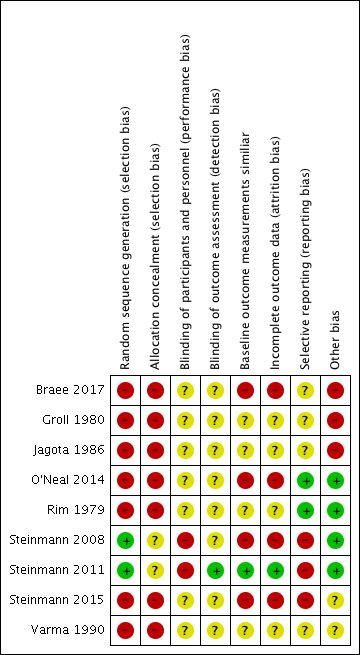

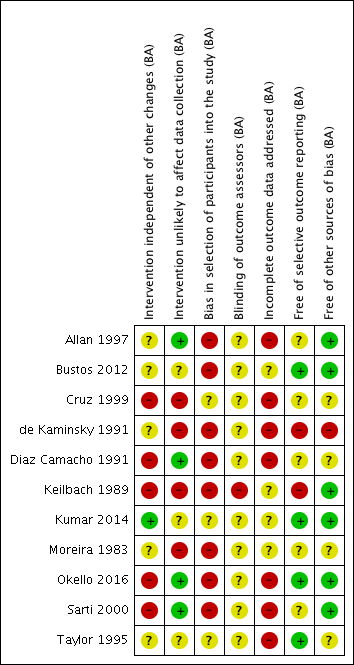

Supplement: S1 Fig — (DOCX) [file pntd.0007873.s006.docx]
