# Supplementary material for: Systematic review of the effectiveness of selected drugs for preventive chemotherapy for Taenia solium taeniasis
Source: PLoS Negl Trop Dis. 2020 Jan 16;14(1):e0007873. doi: 10.1371/journal.pntd.0007873 (PMC6964831; doi:10.1371/journal.pntd.0007873)
Supplement: S2 Fig — Panel A, meta-analysis of cure rate. Panel B, meta-analysis of relative reduction in prevalence. (DOCX) [file pntd.0007873.s007.docx]

## S2 Fig. Doi plots of publication bias for the effect of preventive chemotherapy with different drugs and doses for *Taenia solium* taeniasis. Panel A, meta-analysis of cure rate. Panel B, meta-analysis of relative reduction in prevalence.

**A. Meta-analysis of cure rate**

**B. Meta-analysis of relative reduction in prevalence**
