# Supplementary material for: Systematic review of the effectiveness of selected drugs for preventive chemotherapy for Taenia solium taeniasis
Source: PLoS Negl Trop Dis. 2020 Jan 16;14(1):e0007873. doi: 10.1371/journal.pntd.0007873 (PMC6964831; doi:10.1371/journal.pntd.0007873)
Supplement: S1 Table — (DOCX) [file pntd.0007873.s008.docx]

## S1 Table. Strategies used to diagnose *Taenia solium* taeniasis

| **Study ID** | **Microscopy** | **Macroscopy – scolex, number of uterine branches** | **Copro-Ag** | **Observations regarding approach to *Taenia* *spp.* identification** |
| --- | --- | --- | --- | --- |
| Allan 1990 | Formal-ether concentration (Ritchie 1948) |  | Copro-Ag-ELISA (Allan et al. 1990) | *Taenia* egg identification (microscopy) and Copro-Ag-ELISA were used. Microscopy, as a copro-parasitologic tool, does not allow the distinction between *Taenia* spp. eggs; and the coproantigen detection methodology (Allan et al. 1990) is genus specific, which allows for cross-reactions with other *Taenia* species. Thus, the approach for *Taenia* identification was **non-species specific (NSS).** |
| Braae 2017 |  |  | Copro-Ag-ELISA (Allan et al. 1990) | An attempt to differentiate *T. solium* from *T. saginata* using a copro-Ag-ELISA (Allan et al. 1990) was made, with slight modification (Mwape et al. 2012). The approach was **NSS.** See above. |
| Bustos 2012 |  | Tapeworm scolex identification | Copro-Ag-ELISA (Allan et al. 1990) | Macroscopic search for proglottids and scolexes in feces was performed. Follow-up stool samples were processed for both microscopy and Copro-Ag-ELISA (Allan et al. 1990). There was no proglottid count of uterine branches. The approach was **NSS.** |
| Cruz 1989 | Kato-Katz technique (follow-up only) | Macro examination of participants self-collected tapeworms (baseline) |  | Baseline - 'a plastic bag was given to participants ... to collect the tapeworms expelled.' These were only inspected macroscopically. No further count of proglottid uterine branches was made. Follow-up was based on Kato-Katz examination. The approach was **NSS.** |
| de Kaminsky 1991 | Kato-Katz and scotch tape perianal swab (STPS) techniques | Macro examination of participants self-collected proglottids |  | This study aimed at testing 3 methods to determine the prevalence of *T. solium*: 1) history of proglottid expulsion where participants 'were requested to recover proglottids ... fixed in 10% formalin ... for permanent carmine staining of species identification'; 2) Kato -Katz technique; and 3) STPS. Proglottids and strobila collection were low; no counting of proglottid uterine branches was made; and microscopic techniques (Kato-Katz and STPS) were used. The strategy was **NSS.** |
| Diaz Camacho 1991 | Methods by Faust et al; Ritchie; and Martin and Beaver were used. | Microscopic examination of recovered scolexes and proglottids, including measurement of uterine branches. |  | Microscopic stool examination was carried out using 3 separate methods. *Taenia* spp. positives (eggs) received treatment, followed by castor oil 1h later. Participants were asked to collect their stools (worms) for further analysis (sieving method of Salazar-Chettino and De Haro). Microscopic examination of scolexes, proglottids and counting of uterine branches were performed. Also, serology (ELISA following Larralde et al. 1986) was carried out. The methodology was **species-specific** at baseline**.** |
| Groll 1980 | Egg identification, technique not specified | Proglottid search. No identification of uterine branches. |  | For this study, proglottids and eggs were identified but no methodology is described other than to state: 'stools were examined by well documented techniques according to routine methods…' There was no proglottid count of uterine branches. The methodology was **NSS.** |
| Jagota 1986 | Kato-Katz; STPS and egg count/g of stool |  |  | Fecal examination by the direct method and ova counts by the Kato-Katz method were used as identification techniques. The study methodology was **NSS.** |
| Keilbach 1989 | Coprological examination (egg count), following Faust | Fecal examination for proglottids |  | Coprological examination found 3% (24/760) of participants with taeniasis. Of these, 0.9% expelled proglottids/segments. A differential diagnosis (*T. solium*/*T. saginata*) was made, but no methodology is described. There was no proglottid count of uterine branches. The methodology was **NSS**. |
| Kumar 2014 | Unstained, wet saline mount preparations; Ritchie | Macroscopic identification of scolexes, proglottids, or whole tapeworms. |  | Even though an attempt was made to look for proglottids/scolexes in the samples, there was no species differentiation. The methodology was **NSS.** |
| Moreira 1983 | Sedimentation technique; Ritchie | Proglottid in feces. |  | A differential diagnosis between *T. solium* and *T. saginata* was made based on fecal examination of proglottids, or eggs. There was no count of proglottid uterine branches for further identification of the parasites. **The methodology was NSS.** |
| O'Neal et al. 2014 | Sedimentation technique; light microscopy identification | Scolex, including rostellar hooks identification. Proglottid search. | Copro-Ag-ELISA (Allan et al. 1996) | A comprehensive methodology was followed to establish a diagnosis of *T. solium* taeniasis. In addition to egg count and egg identification (microscopy), taeniid material was recovered, with the subsequent search for rostellar hooks on scolexes and the counting of uterine branches (≤10 branches in gravid proglottids were regarded as *T. solium* positive). Also, serology (EITBrES33) and ELISA tests were carried out. The methodology was **species specific.** |
| Okello 2016 | Microscopy for egg identification | Proglottid identification | Copro-Ag-ELISA (Allan 1990). Copro PCR at the 12S rRNA locus. | A 3-step approach was used to identify *T. solium* in pre- and post-intervention fecal samples: 1) coproantigen identification of taeniid material (Allan and Craig 2006); 2) microscopy for genetic material (eggs/proglottids) search in Copro-Ag positives; and 3) PCR on all microscopy positives. Pre-intervention Copro-Ag-ELISA detected 37 cases of taeniasis (10 microscopy positives); of these 8 and 2 matched sequences of *T. solium* and *T. saginata*, respectively. Microscopy results varied greatly from those of Copro-Ag-ELISA (10/37, 27%). The methodology was **species specific in a sub-sample.** |
| Rim 1977 | Cellophane thick smear and formalin-ether sedimentation methods. | Scolex and proglottid search pre- and post-treatment; also, identification of uterine branches. |  | *T. solium* was identified by macro- and microscopic inspection of taeniid material. Pre- and post-intervention fecal samples were collected (3 consecutive days each). Unfortunately, no scolexes were found and proglottids were destroyed by treatment, precluding uterine branch counting. The methodology was **NSS.** |
| Sarti 2000 | Taenia egg detection (Ritchie 1948). |  | Co-Ag-ELISA (Allan et al. 1990) | Pooled results (coproantigen and egg detection) at T0 showed 16 cases of taeniasis diagnosed by Copro-Ag-ELISA, 11 by egg detection, and 6 by both methods. Similar discrepancies between the two methods were found at T1 and T2. The Co-Ag-ELISA test was based on Allan et al. 1990 and no other form of *T. solium* identification took place. The methodology was **NSS**. |
| Steinmann 2008 | 3 microscopy methods: Eggs/g (Kato-Katz); Koga agar plate; and Bearmann test |  |  | In this study, microscopy for egg count and egg and larvae identification were used. The methodology was **NSS.** |
| Steinmann 2011 | Kato-Katz thick smear technique | Visual inspection of proglottids |  | The primary outcomes for this study were cure rate/egg reduction rate. The focus was on proglottid recovery and egg identification of *Taenia* spp. The methodology was **NSS.** |
| Steinmann 2015 | 3 microscopy methods: Eggs/g (Kato-Katz); Koga agar plate; and Bearmann test | Visual inspection of Taenia sp. proglottids |  | A visual inspection of proglottids and 3 microscopy techniques were used as identification methods. The methodology was **NSS.** |
| Taylor 1995 | Microscopy for egg identification |  |  | Microscopy was used for egg identification, but the authors do not specify by what technique, nor did they attempt to establish any species differentiation. The methodology was **NSS.** |
| Varma 1990 | Microscopy for egg identification (technique no mentioned) | Tapeworm strobila/scolex recovery |  | This study aimed at establishing cure rate following treatment. The absence of taeniid material (scolexes, strobila or eggs) on samples was regarded as cure. The methodology was **NSS.** |

NSS - non-species specific; STPS - scotch tape perianal swab; MDA – mass drug administration

**References**

Allan JC, Avila G, Garcia Noval J, Flisser A, Craig PS. Immunodiagnosis of taeniasis by coproantigen detection. Parasitology 1990;101 Pt 3: 473-7.

Allan JC, Velasquez-Tohom M, Torres-Alvarez R, Yurrita P, Garcia-Noval J. Field trial of the coproantigen-based diagnosis of Taenia solium taeniasis by enzyme-linked immunosorbent assay. Am J Trop Med Hyg 1996;54: 352-6.

Mwape KE, Phiri IK, Praet N, Muma JB, Zulu G, Van den Bossche P, et al. *Taenia solium* Infections in a rural area of Eastern Zambia - a community based study. PLoS Negl Trop Dis 2012;6: e1594.

Ritchie LS. An ether sedimentation technique for routine stool examinations. Bull U S Army Med Dep 1948;8: 326.
