# Supplementary material for: Systematic review of the effectiveness of selected drugs for preventive chemotherapy for Taenia solium taeniasis
Source: PLoS Negl Trop Dis. 2020 Jan 16;14(1):e0007873. doi: 10.1371/journal.pntd.0007873 (PMC6964831; doi:10.1371/journal.pntd.0007873)
Supplement: S2 Table — A. Results of the studies that tested mass drug administration (with or without selective chemotherapy). B. Results of the studies that tested selective chemotherapy . (DOCX) [file pntd.0007873.s009.docx]

## S2 Table. Infection rate with *Taenia solium* taeniasis

## Table A. Results of the studies that tested mass drug administration (with or without selective chemotherapy)

| **Study ID** | **Country** | **Study Design** | **Drug** | **Dose** | **Time of follow-up (months)** | **Prevalence at baseline (n/N)** | **Prevalence at follow-up (n/N)** | **Total N** | **Relative reduction in prevalence** |
| --- | --- | --- | --- | --- | --- | --- | --- | --- | --- |
| Allan 1997 | Guatemala | BA | NICL | 2g | 10 | 3.54% (56/1582) | 1.0% (11/1116) | 1116 | 72% |
| Braae 2017 Annual MDA children | Tanzania | CBA | PZQ, NICL, PZQ | 40mg/kg | 9-10 | 2.31% (22/951) | 0.1% (1/1070) | 951 | 96% |
| Biennial MDA children |  |  | PZQ, NICL, PZQ | 40mg/kg | 9-10 | 1.25% (11/880) | 0.4% (4/1076) | 880 | 70% |
| SC adults (annual MDA) |  |  | NICL, PZQ | 2g, 10mg/kg | 9-10 | 4.10% (23/561) | 1.8% (8/444) | 444 | 56% |
| SC adults (biennial MDA) |  |  | NICL, PZQ | 2g, 10mg/kg | 9-10 | 1.77% (11/621) | 0.7% (3/425) | 425 | 60% |
| Cruz 1989 | Ecuador | BA | PZQ | 5mg/kg | 12 | 1.55% (148/9529) | 0.0% (0/539) | 539 | 100% |
| Diaz Camacho 1991 | Mexico | BA | NICL, PZQ | 2g, 10mg/kg | 12 | 1.32% (4/302) | 0.0% (0/283) | 283 | 100% |
| Sarti 2000 ^a^ | Mexico | BA | PZQ | 5mg/kg | 42 | 1.13% (21/1865) | 0.5% (3/605) | 605 | 56% |
| Steinmann 2008^b^ | China | RCT | ALB | 400mg | 1 | 40.91% (27/66) | 25.8% (17/66) | 66 | 37% |
| Steinmann 2011  single dose^c^ | China | RCT | ALB | 400mg | 1 | 13.41% (11/82) | 7.3% (6/82) | 82 | 45% |
| triple dose^d^ |  |  | ALB | 3x400mg | 1 | 10.29% (7/68) | 0.0% (0/68) | 68 | 100% |
| Steinmann 2015   Annual MDA^e^ | China | CBA | ALB | 400mg | 24 | 14.80% (14.8/100) | 6.7% (6.7/100) | 100 | 55% |
| 6-monthly MDA^e^ |  |  | ALB | 400mg | 24 | 13.70% (13.7/100) | 10.6% (10.6/100) | 100 | 23% |

ALB - albendazole; BA - before-after study; CBA - controlled before-after study; MDA – mass drug administration; NICL - niclosamide; PZQ - praziquantel; RCT - randomized controlled trial; SC – selective chemotherapy

a – Prevalence at the 6-month follow-up was 0.53% (7/1311), with a relative reduction in prevalence of 53%

b – 10 ‘new’ positive infections at follow-up were added to the number positive at baseline. Cure rate: 37% (10/27)

c – 1 ‘new’ positive infection at follow-up was added to the number positive at baseline. Cure rate: 45% (5/11)

d – Cure rate: 100% (7/7); Relative risk 2.2 (95%CI 0.7-3.7) for cure with triple dose versus single dose ALB 400mg.

e – Results at 2-year follow-up; Total N of 100 is approximate

## Table B. Results of the studies that tested selective chemotherapy

| **Study ID** | **Country** | **Study Design** | **Drug** | **Dose** | **Time of final follow-up (days)** | **Total N** | **N cured** | **Cure rate** |
| --- | --- | --- | --- | --- | --- | --- | --- | --- |
| Bustos 2012 | Peru | BA | NICL | 2g | 90 | 68 | 53 | 77.9% |
| de Kaminsky 1991 | Honduras | BA | ALB | 3x400mg | 90 | 56 | 56 | 100.0% |
| Groll 1980 | Various | CBA | PZQ | 10mg/kg | 90 | 33 | 33 | 100.0% |
|  |  |  | PZQ | 5mg/kg | 90 | 9 | 6 | 66.7% |
| Jagota 1986 | India | CBA | ALB | 400mg | 21^a^ | 37 | 24 | 64.9% |
|  |  |  | ALB | 3x400mg | 21^a^ | 37 | 32 | 86.5% |
| Kumar 2014 | India | BA | PZQ | 10mg/kg | 14-21 | 57 | 57 | 100.0% |
| Moreira 1983 | Brazil | BA | PZQ | 10mg/kg | 90 | 31 | 31 | 100.0% |
| Rim 1979 | Korea | CBA | PZQ | 10mg/kg | 90 | 27 | 27 | 100.0% |
|  |  |  | PZQ | 5mg/kg | 90 | 26 | 25 | 96.2% |
| Taylor 1995 | South Africa | BA | PZQ | 40mg/kg | 45 | 3 | 3 | 100.0% |
| Varma 1990 | India | CBA | NICL | 2g | 90 | 38 | 36 | 94.7% |
| O'Neal 2014^b^ | Peru | CT | NICL | 2g | 120 (after final treatment) | 0.7% (5/703) intervention vs  1.6% (9/555) control, adjusted prevalence ratio 0.28 (95%CI 0.08-0.91) | | |

ALB - albendazole; BA - before-after study; CBA - controlled before-after study; CT - controlled trial; NICL - niclosamide; PZQ - praziquantel

a – Some participants were also tested at 90 days post-treatment

b – Figures are prevalence of confirmed taeniasis at follow-up
