# Supplementary material for: Systematic review of the effectiveness of selected drugs for preventive chemotherapy for Taenia solium taeniasis
Source: PLoS Negl Trop Dis. 2020 Jan 16;14(1):e0007873. doi: 10.1371/journal.pntd.0007873 (PMC6964831; doi:10.1371/journal.pntd.0007873)
Supplement: S3 Table — (DOCX) [file pntd.0007873.s010.docx]

## S3 Table. Data extracted from included studies on the outcomes: costs, cost-effectiveness, feasibility, values and preferences of participants, and impact on equity (n=5)

| **Study ID & country** | **Intervention^a^** | **Costs, cost-effectiveness** | **Feasibility** | **Values and preferences of participants** | **Impact on equity** |
| --- | --- | --- | --- | --- | --- |
| Cruz 1989  Ecuador | MDA PZQ - 5mg/kg body weight | The average cost of treating an adult was US$0.30 at the 1986 retail price of praziquantel to United Nations agencies. In Ecuador the average cost of the drug to treat one case of human cysticercosis was estimated to be US$ 187, and in Mexico in 1982 the average cost of hospitalizing a patient with the disease was US$2173.8. By law, infected pigs are usually destroyed, which means the loss of US$ 50-150 per animal depending on the weight at slaughter. | "The operational studies reported ... confirm the feasibility of using largescale chemotherapeutic intervention." | "The treatment was well accepted by the local population. The socio-anthropological study in Gonzanama revealed that 90% of the people who had been treated and interviewed were interested in the continuation of the project. Only in one settlement, where the information campaign was not particularly active, and one of the local leaders opposed the project, did 50% of the population interviewed show no interest in further participation." | The distribution of taeniasis was statistically dependent on household size (7.1% of households with seven or more people had one or more carriers) but was not dependent on economic or sanitary indices such as the type of house, water source, toilet availability, and whether pigs were bred at home (χ^2^ test, P > 0.05). |
| Keilbach 1989  Mexico | MDA PZQ - 5mg/kg body weight |  | Despite promised support from health authorities to provide human resources and housing most of this did not materialize beyond the first week of the trial. Due to lack of education amongst the adults it was hard to make them understand health messages and they showed a strong reluctance to give up traditional beliefs. However, children were enthusiastic and eager to learn. | Only about 60% of inhabitants agreed to take the PZQ. "One of the explanations is lack of interest and mistrust. As a rule, men showed less interest than women in the programme and were reluctant to cooperate. Few attended the meetings and most refused to bring their stool samples or let their blood be taken. Mistrust was a widespread factor in the community and many suspected some sort of business with their blood." |  |
| Okello 2016  Lao People’s Democratic Republic | MDA ALB - 400mg per day for three consecutive days |  | The use of praziquantel was not permitted by the Lao PDR Ministry of Health. | "Compliance with MDA was high due to trust between project staff and village leaders. However, understandings of pharmacology, minor side effects, human migration and children's fear of worms may lead to resistance in future programmes." "concern involved compliance of school-aged children, as related to an existing school deworming programme (part of a long-standing donor-funded nutrition project) where all students in the district receive a biannual dose of Mebendazole 500mg (the project intervention used three consecutive 400mg doses of Albendazole)." "a number of parents did not consent to their school-aged children taking antihelmintics during the project-led MDA, citing the existing school programme as sufficient." However, "The local schoolteachers ... estimating that only 35 percent of children were regularly ingesting these tablets." |  |
| Steinmann 2011  China | MDA ALB - single-dose (400mg) or triple-dose (3x400mg) |  |  | "From a patient perspective, triple dose treatment appeared acceptable in the present study." |  |
| Taylor 1995  South Africa | Selective chemotherapy PZQ - 40mg/kg body weight |  | "The inaccessibility of some of the creches precluded repeated visits by the researchers for follow-up purposes." |  |  |

Abbreviations: ALB - albendazole; MDA - mass drug administration; PZQ - praziquantel; NR - not reported; SES - socioeconomic status;

a - all studies conducted in all age groups, except for Taylor et al. 1995, which was conducted in pre-school children 4-6 years of age.
