# Supplementary material for: Systematic review of the effectiveness of selected drugs for preventive chemotherapy for Taenia solium taeniasis
Source: PLoS Negl Trop Dis. 2020 Jan 16;14(1):e0007873. doi: 10.1371/journal.pntd.0007873 (PMC6964831; doi:10.1371/journal.pntd.0007873)
Supplement: Attachment — Submitted filename: PNTD-D-19-01307 Response to reviewers comments.docx [file pntd.0007873.s011.docx]

**"Systematic review of the effectiveness of selected drugs for preventive chemotherapy for Taenia solium taeniasis" (PNTD-D-19-01307) - PLOS Neglected Tropical Diseases**

**Responses to Reviewers’ Questions/Comments**
We thank the reviewers for their careful and helpful review of our manuscript. Our responses to the reviewer comments follow each comment (underlined).

Methods
Reviewer #1: see below. - No response required here.

Reviewer #2: The study, considering its type, follow clear and concrete objectives and the methods are in good articulation with these objectives. The only thing that I would add to the explanation of the methods used is the qualifications, competences and language skills for the two independent reviewers – We have never seen a systematic review that published these kinds of details in the methods section of the review. Further, we feel uncomfortable adding the details as the review was conducted by a multidisciplinary team (as can be seen by the author list). While the two independent reviewers made the majority of decisions about inclusion or not, they were guided by the third reviewer and other authors. However, we have added the initials of the three main reviewers to the manuscript (pages 12 and 13).

Reviewer #3: The methodology that was used for this systematic review is of good quality; among other things, PRISMA checklist and flow diagram are included; 2 reviewers performed the searches and screening and several electronic databases were used. - No response required here.

--------------------

Results
Reviewer #1: see below - No response required here.

Reviewer #2: Results are presented in a clear and complete form, and the tables show data in a suitable way – Thank you. No response required here.

Reviewer #3: The data are presented well and the authors are open about the papers/data that were used. – Thank you. No response required here.

--------------------

Conclusions
Reviewer #1: see below - No response required here.

Reviewer #2: Conclusions are straight and supported by the evidence shown. Limitations or potential bias are clearly stated. The public health relevance of the study and its results are addressed correctly - No response required here.

Reviewer #3: The authors are generally clear about the limitations and uncertainties that remain. The general picture and public health relevance is clearly addressed. - No response required here.

--------------------

Editorial and Data Presentation Modifications?

Reviewer #1: see below - No response required here.

Reviewer #2: Use always italics when writing scientific names, even in the abstract, at the beginning of the manuscript – We searched carefully through the manuscript (using the find tool in Word) for all mentions of *T. solium* or *Taenia solium* and updated a few that weren’t already in italics.

In line 36: It would be recommendable to give more details on PROSPERO – We have added the full name of the register. We also modified the wording a little to conform with that suggested by PROSPERO.

In line 55: Consider the inclusion of a comma: ....are considered, by the WHO, to be neglected.... – We added the two commas as suggested (now line 57)

In line 59: I do not consider that it is totally true that after years of treating people it is not known the best drug and the most appropriate dose are completely, I think that the statement could be changed to reflect in a better way what is the status on this area and what it still to be explore and assessed.

It has been changed now to better reflect the situation.

In lines 86-87: I think that the redundant reference to seizures could be modified to avoid repetitions of the same idea. We respectfully disagree that the reference to seizures is redundant in the statement: “Cerebral cysts are a major cause of adult onset seizures in most low-income countries [3].” because it is the first time that seizures are mentioned in the paper. Further, the statement shows the impact of the condition in low-income countries.

In line 103: I do not find useful the reference to "traditional medicine" as the goal of the work has nothing to do with this topic – We have deleted the reference to traditional medicine.

In line 107: Correct the term "inn", it should read "in" – Thank you for spotting this. It has been corrected.

In line 115: Avoid using italics in writing "spp" – We have checked the entire manuscript and corrected the use of italics for spp.

In line 138: Include the term PROSPERO to be correspondent to the abstract and summary – Done

In line 170: I am not fully convinced of the use for the term "copro-DNA", perhaps it could be changed to "molecular techniques" or "use of genomic markers" or something similar. – Copro-DNA is used to include several molecular techniques that can be used in faeces, and it is a term commonly used in the field, therefore it has not been changed.

Reviewer #3: (No Response)

--------------------

Summary and General Comments

Reviewer #1: I enjoyed very much reading this manuscript. Thank you. We’re glad that you liked the paper. I have several comments and recommendations that I hope the authors will follow.

In lines 39 - 41 praziquantel should go before albendazole, so that efficacies can be listed from higher to lower, the same is true for lines 41-42, for figure 3 and for lines 493-494. – We have changed the order of praziquantel and albendazole, along with their respective efficacies in lines 40-44 and lines 500-4. We also made the same change in the rest of the paragraph for consistency (paragraph 2 of Discussion). We prefer not to change the order in figure 3 as it would require a major re-organization of the data in Excel and replotting of the figures, which we fear could result in inadvertent errors.

Line 62 where should be when – Done

Line 184 are should be changed for have – We disagree with the reviewer but are happy for the Editor to decide. The sentence is: “Case reports were excluded because they are a very low level of evidence of effect and chance cannot be ruled out.”

Line 266 I think that in sufficient should be insufficient – We agree and have corrected this error. Thank you.

Line 272 I think the search performed by the authors deserves an explanation of the reasons why 3299 records were removed. – We have added an explanation as to why the 3299 records were removed. This process is standard for systematic reviews, as explained in the methods (Study Selection, pages 12-13) and is shown explicitly in Fig 1.

Line 357 the authors should give a valid explanation to the fact that the only study that used a species-specific diagnostic test was not included because of "different outcome measure". –This study could not be included in the **meta-analysis** due to the fact that it did not measure either cure rate or relative reduction in prevalence. This has been clarified now (lines 361-362). Please note that the paper wasn’t excluded completely from the review. The results of the study are reported in Table S2 and are also noted on page 22 of the manuscript: “… and the other was a controlled trial of ring screening followed by selective chemotherapy, showing that the intervention resulted in a lower prevalence of *T. solium* at follow-up (adjusted prevalence ratio 0.28, 95%CI 0.08-0.91) compared to no intervention [45].”

Line 373 could the authors please specify why PZQ at 10 mg/ml "tended to give better results" – we added the statement “(based on comparison of the pooled cure rates and 95%CI)” to the sentence. We hope that this makes it clearer. A visual inspection of Figure 3 also supports this statement.

Lines 415 and 416 have duplicated information, I suggest eliminating that of line 416. – we have removed the duplicated information.

The first paragraph of the discussion (lines 486-491) does not mention the word taeniasis and I think this is an important absence. – We agree and have added *T. solium* taeniasis to the second sentence.

Line 537 Schistosomiasis should be without a capital letter – Thank you. We have corrected this error.

Line 563 "one included study (45), but in line 357 it states that this study was not included. – This study (45) was included in the systematic review. We specified in line 357 (now line 362) that it was not included in the **meta-analysis**. However, as clarified above, it was included in the narrative synthesis of the evidence.

I don't agree with the authors that conclude with a recommendation (lines 585-593) that more studies should be done to evaluate triple dose ALB 400mg, PZQ 5 vs 10mg/kg, NICL 2g; they convinced me that all, except PZQ at 5mg are good choices and, in endemic areas, niclosamide would be selected. – In this recommendation we state that high quality randomized controlled trials are needed. We go on to clarify, in this same paragraph, the methodological issues that should be addressed in such trials. More research is necessary because all included studies had a high risk of bias and few randomized controlled trials have been done. Thus, the body of evidence for each outcome has a low (or very low) certainty. Thus, better research is needed to satisfy the current standards for WHO Guidelines (reference 19).

I think that references 37 and 58 are the same study; the first one is described in English, the second one in Spanish. – Yes, we agree. This is why both references are cited in the Tables and main text (paragraph 1 of the results) when citing the Cruz et al. 1989 study. Both references were found by the search strategy and selected for inclusion. However, given that they were identified as reporting the same study, the English version (37) is denoted as the primary reference to the study and the Spanish version (58) is denoted as the secondary reference as it was published the following year.

Table 3 lacks countries; it would be useful to include this information – We have added country information to Table 3 as requested.

Table 4 last line of the first column, the word India should be added – Done.

Figure 1 first square of records excluded, should have a brief explanation – Further explanation was added to the first paragraph of the results section. We also added the words “(titles and abstracts)” to the box: “Records screened” in Figure 1. We hope that this is now clearer. Also, to further clarify, Figure 1 uses the template provided as part of the PRISMA statement (reference 18) and can be found here: <http://www.prisma-statement.org/PRISMAStatement/FlowDiagram>.

Figure 1 second square, the explanation of full text articles "with reasons", is absurd, or you add an adequate explanation or you delete the one used – As noted in the previous comment, the Figure uses the template provided as part of the PRISMA statement (reference 18) and can be found here: <http://www.prisma-statement.org/PRISMAStatement/FlowDiagram>. The wording used is standard for systematic reviews. The reasons for exclusion of each of these 221 articles is given in S3 file, which is referenced in the first paragraph of the results section.

Figure 2 panel B, third line says slection, should be selection – Thank you for picking this up. We have corrected this spelling error. We also corrected the same error in S1_Fig, which is generated from the same data.

Congratulations – Thank you. We are very glad that you liked the paper. Thank you also for revising it so carefully.

Reviewer #2: In general this manuscript constitutes a very interesting and well written work, with clear objectives, which are scientifically justified, and clear results. – Thank you. We appreciate your comments. Only some minor modifications suggested could be incorporated to further improve the quality of the document

Reviewer #3: L250: ‘infection rate’ doesn’t seem to be the correct term here since you mention that cure rate/reduction is usually used. – When we started the review, we used the term ‘infection rate’ in the protocol as we weren’t sure exactly what measures would be presented. Thus, the use of this term here is to be consistent with the protocol and is also consistent with the methods section (which is based on the protocol) – see Outcome measures, now page 10.

L457: so what are the conclusions of these studies [46 and 49] – We have added a reference to Table 4 in this paragraph to make it clearer that the results of these studies are in that table. Note: Table 4 is first cited in the previous paragraph.

L493-500: not finding a difference doesn’t mean there is no difference (L496-497). The number of studies was extremely low to do a proper evaluation. This should be clearly mentioned. – We have added the sentence: “However, it should be noted that the lack of statistical significance does not mean that there is no difference but could be due to the small number of studies.”

L550-552: this is not very clear. There was no difference for children < 12="" _years2c_="" but="" in="" l552="" it="" is="" mentioned="" ages="" 3-79="" years. – We have clarified the sentence to state: “A comprehensive, multi-country randomized placebo-controlled trial (n=870, ages 3-79 years) found no significant difference in the number of side-effects between ALB 400mg (children <12 years were given 200mg) and placebo [80].” We hope that this is now clearer.

="" figure="" _13a_="" the="" additional="" records="" are="" 23="" _figure2c_="" seems="" to="" be="" different="" from="" number="" s1="" file.="" _22_full-text="" articles="" _excluded2c_="" with="" _reasons22_3a_="" which="" _reasons3f_="" include="" reasons.="" diagnosis="" of="" taeniosis="" very="" difficult.="" this="" should="" emphasized="" more="" manuscript="" as="" a="" major="" limitation="" for="" proper="" evaluation="" drugs="" effectiveness.="" methodology="" that="" was="" used="" _figures2f_tables="" where="" possible.="" _l592-5933a_="" test="" would="" you="" _suggest3f_="" --------------------="" - – We are unable to understand what the reviewer’s comment is here.
