# Supplementary material for: Systematic review of the effectiveness of selected drugs for preventive chemotherapy for Taenia solium taeniasis
Source: PLoS Negl Trop Dis. 2020 Jan 16;14(1):e0007873. doi: 10.1371/journal.pntd.0007873 (PMC6964831; doi:10.1371/journal.pntd.0007873)
Supplement: Attachment — Submitted filename: PNTD-D-19-01307R1 Response to reviewers comments.docx [file pntd.0007873.s012.docx]

**"Systematic review of the effectiveness of selected drugs for preventive chemotherapy for Taenia solium taeniasis" (PNTD-D-19-01307R1) - PLOS Neglected Tropical Diseases**

**Responses to Reviewers’ Questions/Comments**
We thank the reviewers for their careful and helpful review of our manuscript. Our responses to the reviewer comments follow each comment (underlined).

Reviewer #1: The authors responded to all my comments and made the necessary changes - No response or action required here.

Reviewer #2: No response - No action required here.

Reviewer #3: There seem to have been a technical problem for my last comments to the first submission, so here they are again:

Figure 1: the additional records are 23 in the figure, but seems to be different from the number in S1 File. – Thank you for picking up this error, which we have checked carefully. The figure is correct. The error is in S1_File (page 2), which we have now updated to clarify that 23 records were picked up in the extra searches.

The diagnosis of taeniosis is very difficult. This should be emphasized more in the manuscript as it is a major limitation for the proper evaluation of drugs effectiveness. Include the methodology that was used for diagnosis of taeniosis in the figures/tables where possible.

We believe that paragraph 570-580 (in the clean version of the submitted reviewed manuscript) clearly conveys the message. Further, the manuscript already includes the methodology for diagnosis in S1_Table and the issues are presented in the methods section (lines 170-178) and results (lines 355-361). Thus, we believe that what is at issue here has been sufficiently emphasized throughout the paper.

L592-593: which test would you suggest?

Unfortunately, we are not in the position to recommend any test.  As the reviewer has correctly pointed out above ‘the diagnosis is taeniasis is very difficult’. There is only one copro-antigen that has been published to be both sensitive and specific (Guezala et al, 2009), but it is not commercially available and is not that easy to validate in-house.  There are many molecular tests described, including conventional PCR, real time PCR, and LAMP, but they have not been independently or appropriately validated. Some of the most promising ones are the nested PCR published by Mayta et al, 2008, and the LAMP test published by Nkouawa et al, 2010.   For more details, we refer the reviewer to Reference 20 in the manuscript (Lightowlers et al, 2016).
